# A generative growth model for thalamocortical axonal branching in primary visual cortex

**DOI:** 10.1371/journal.pcbi.1007315

**Published:** 2020-02-13

**Authors:** Pegah Kassraian-Fard, Michael Pfeiffer, Roman Bauer

**Affiliations:** 1 Institute of Neuroinformatics, University and ETH Zurich, Zurich, Switzerland; 2 Division of Biology and Biological Engineering, California Institute of Technology, Pasadena, California, United States of America; 3 Interdisciplinary Computing and Complex BioSystems Research Group (ICOS), School of Computing Science, Newcastle University, Newcastle upon Tyne, United Kingdom; 4 Biosciences Institute, Newcastle University, Newcastle upon Tyne, United Kingdom; The University of Queensland, AUSTRALIA

## Abstract

Axonal morphology displays large variability and complexity, yet the canonical regularities of the cortex suggest that such wiring is based on the repeated initiation of a small set of genetically encoded rules. Extracting underlying developmental principles can hence shed light on what genetically encoded instructions must be available during cortical development. Within a generative model, we investigate growth rules for axonal branching patterns in cat area 17, originating from the lateral geniculate nucleus of the thalamus. This target area of synaptic connections is characterized by extensive ramifications and a high bouton density, characteristics thought to preserve the spatial resolution of receptive fields and to enable connections for the ocular dominance columns. We compare individual and global statistics, such as a newly introduced length-weighted asymmetry index and the global segment-length distribution, of generated and biological branching patterns as the benchmark for growth rules. We show that the proposed model surpasses the statistical accuracy of the Galton-Watson model, which is the most commonly employed model for biological growth processes. In contrast to the Galton-Watson model, our model can recreate the log-normal segment-length distribution of the experimental dataset and is considerably more accurate in recreating individual axonal morphologies. To provide a biophysical interpretation for statistical quantifications of the axonal branching patterns, the generative model is ported into the physically accurate simulation framework of Cx3D. In this 3D simulation environment we demonstrate how the proposed growth process can be formulated as an interactive process between genetic growth rules and chemical cues in the local environment.

## Author summary

The morphology of axonal arborizations is highly variable and complex, yet the canonical regularities of the cortex imply that axonal branching patterns have developed based on a small set of simple rules. Extracting such growth rules is fundamental, as it can shed light on the genetically encoded instructions for cortical developmental, necessary for subsequent cortical function. We propose in this work a generative model for branching patterns of thalamic afferents in cat area 17, fundamental for the synaptic connections of the ocular dominance stripes, and for a conservation of receptive field properties. The model is optimized for the segment-length distribution as well as for a length-weighted asymmetry quantification of the axonal morphologies, and can hence capture global morphological properties of the entire dataset, as well as morphologies of individual axons. We can show that our mechanistic model clearly surpasses the statistical accuracy of the Galton-Watson model, the most employed model for biological growth processes, a result which underlines the plausibility of the proposed growth rules. Our model is implemented in MATLAB as well as ported into Cx3D, a physically realistic simulation environment, where biophysical interactions with the environment are incorporated in the growth process to shape the final axonal morphology.

## Introduction

Thalamocortical arborizations in the primary visual cortex must be organized with high precision to enable dense synaptic connections for the ocular dominance columns [1, 2] and to preserve the spatial resolution of the associated receptive fields [3]. Arborizations of these thalamocortical axons in cat area 17 are characterized by a high bouton density and extensive and highly variable ramifications, ranging from structures consisting of only a single branch to elaborate morphologies with tens of segments of highly diverse lengths [4]. What developmental rules give rise to such morphological variability while enabling simultaneously the precise wiring required for accurate functionality?

Despite their morphological complexity, local cortical regularities [5] suggest that generic developmental rules enclosed in only few precursor cells might give rise to the thalamocortical arborizations. In line with previous work on the canonical regularities of cortical structure [5–8], we assess here the possibility that these elaborate thalamocortical arborizations are given rise to by the repeated initiation of a small set of genetically-encoded rules, carried out in interaction with the local environment during cortical self-construction [9–13]. This account is furthermore in line with an activity-independent development of the axonal arborizations of the ocular dominance columns [14].

To determine the developmental rules for thalamocortical arborizations underlying the ocular dominance columns, we propose here a generative growth model benchmarked against biological data from layers 4 and 6 of cat area 17. An accurate recreation of axonal morphologies based on a mechanistic modelling approach allows us to shed light on genetically encoded instructions during cortical development [15], and ultimately provides a basis for the empirical assessment of model predictions.

The morphology of axonal arborizations is highly variable and an essential feature for cell type classification [16]. Because of this high variability, however, models recreating axonal arborizations have been few in number. Past models have so far been based on the simple Galton-Watson branching process [9, 17]. The Galton-Watson model [18], the best established and most studied growth model, allows at each step for either bifurcation, growth or halting, all with constant probabilities during the entire growth process. It does, however, not incorporate biophysical constraints, and generates an exponential distribution of segment-lengths. The failure to match this crucial metric for neurites with other segment-length distributions reveals the limits of this growth model.

Other models of neurite growth have been mostly dedicated to dendritic outgrowth [19–24]. Classical dendrite growth models have assumed distinct growth rules depending on the growth cones’ position in the topology of the neurite [19–21], an assumption opposed to biological principles of local autonomy [25]. Further approaches have focused on optimization principles, inspired for instance by Cajal’s conservation principles of cytoplasmic volume, space and conduction time [22, 26]. Such models have generated diverse types of realistic dendrite morphologies, for instance by combining constraints on the total wiring length with constraints on the path length to the root of the dendrite [22]. These global optimization requirements stand, however, in contrast to biological principles of local autonomy, or, alternatively, operate on a descriptive level which does not allow for insights into mechanistic underpinnings of the neurite growth process.

Our approach in contrast pursues a mechanistic account of neurite outgrowth based on local actors, and is in line with more recent generative neurite growth models where the impact of environmental cues on the neurite morphology is taken into account [27]. In particular, the here presented model makes use of simple developmental processes such as growth, bifurcation and retraction. The model is optimized for the segment-length distribution, a quantification based on the entirety of the data, as well as a length-weighted asymmetry index which quantifies the morphology of individual arborization patterns. This newly introduced length-weighted asymmetry index extends on the classical asymmetry index [28, 29] to include metric properties, allowing for a finer control of morphological asymmetries.

In a further step, we port the generative model into Cx3D, a simulation environment for cortical development based on principles of cortical self-construction [30]. This translation allows us to provide a biophysical interpretation of the observed statistics and enables a future integration of the algorithm into broader simulations of cortical development. Within the Cx3D framework, we model the morphological asymmetries as the result of interactions of the growth process with chemotaxic cues, known to be crucial for axonal path finding [31–33] in the local environment.

## Materials and methods

### Dataset

**Fig 1:**
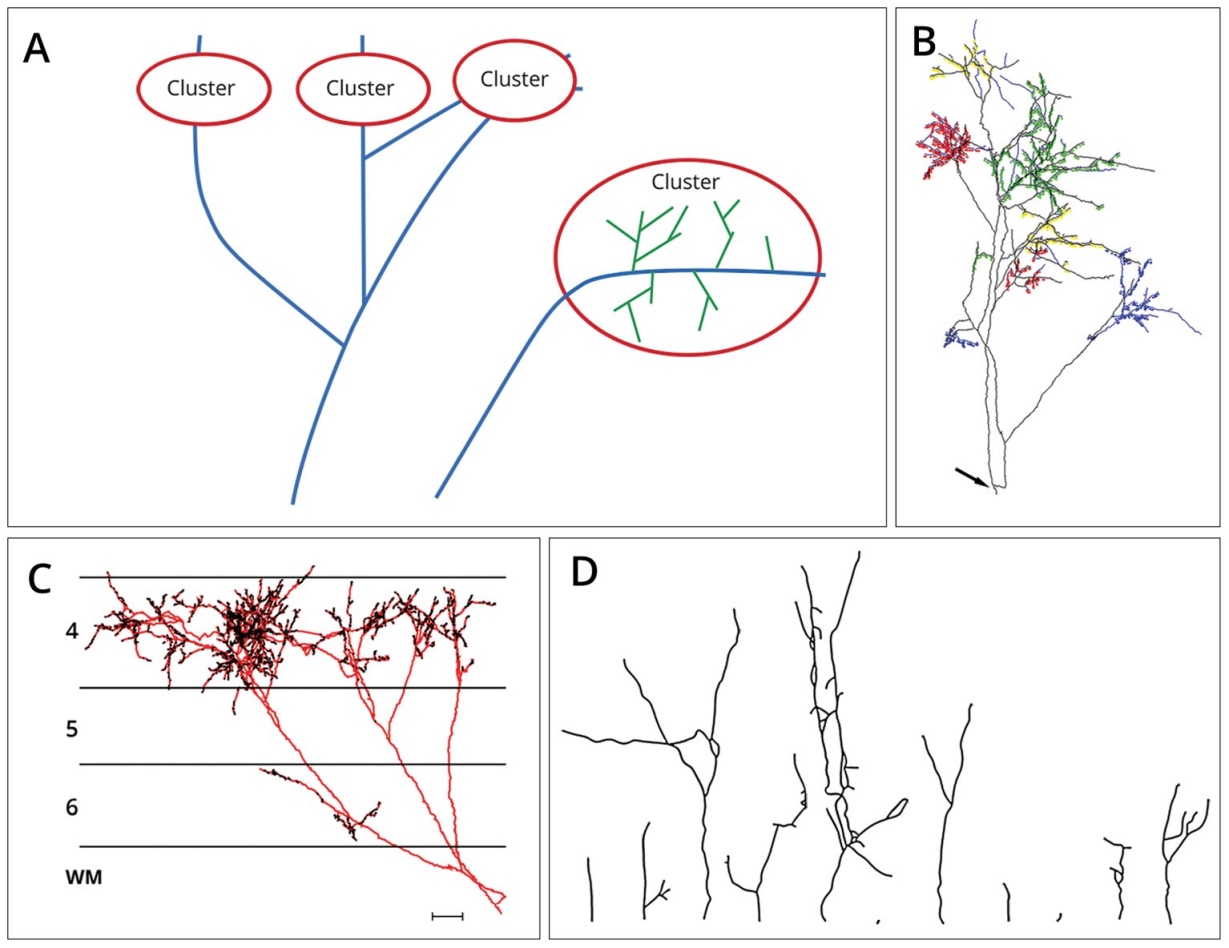
Thalamocortical arborizations in cat primary visual cortex. (A) Clusters containing florets (green) and long-range axonal projections (blue). Besides a higher branching probability than present at the axonal trunk, florets can be characterized by a higher bouton density. The long-range axonal parts outside of the clusters in contrast display fewer branching occurrences, longer branches, and a lower bouton density [4, 10]. (B) Labeled Y axon terminals of cat primary visual cortex arborising in layers 4 and 6. The axon was reconstructed from serial sections and has been rotated to optimize the view of the coloured florets. The axon emerges from the white matter at the location indicated by the arrow. (C) Displayed is the same axon (red) as in (B), here however from a coronal view. The axon emerges from white matter, here indicated by ‘WM’. The terminals of the axon focussing on layers 4 and 6 are indicated in black. A horizontal 100 micron scale bar appears in the bottom right hand corner. (D) Florets aligned by their root segments. Floret morphology is highly variable, with florets consisting of only a single segment (‘trivial florets’) as well as larger florets with many and highly diverse segments being present in the dataset. All experimental data is courtesy of Kevan A.C. Martin and colleagues, Institute of Neuroinformatics, Zurich.

The neurons examined in this study were obtained from anesthetized adult cats that had been prepared for in vivo intracellular recording. All experiments were carried out by Kevan A.C. Martin and colleagues under the authorization of animal research licenses granted by the Home Office of the U.K. and the Cantonal Veterinary Authority of Zurich. A total of 426 axonal arbors from 10 thalamic afferents was collected from 5 adult cats. All axons originate from the dorsal part of the lateral geniculate nucleus and project to layers 4 and 6 of area 17. Axons were classified as X or Y-type using a battery of tests [34–36]. The axons have been used in previous studies [3, 8–10, 34, 36, 37]. Surgical details are found in [8, 36]. After labelling the axons in the anaesthetized cat in vivo using intracellular injections of horseradish peroxidase (HRP) or anterograde tracer biotinylated dextran amine (BDA), the axons were reconstructed from blocks of histologically prepared and serially sectioned tissue. Using a x40 or x100 oil immersion objective attached to a light microscope and drawing tube the axons were reconstructed by using either the in-house 3D reconstruction system TRAKA or Neurolucida. The reconstructions were stored as a list of data points for further usage. Clustered axonal arbors are shown schematically in Fig 1 panel **A**. Axonal arbors from a single thalamocortical afferent branching in layer 4 of the visual cortex are shown in Fig 1 panel **B**. Fig 1 panel **C** shows various thalamic aligned by their root segment. We define the segment length as the length of the neurite between branching points, or between branching points and the tip of the neurite, measured in microns [*μm*] in 3D space. Among the 426 reconstructed arbors, 260 are trivial structures, consisting of a single segment.

### Definition of florets

Due to their morphological difference, we distinguish here between the long-range axonal projections with a relatively low bifurcation ratio which constitute the axonal trunk [4], and the self-similar ramifications in the target area of axonal projections, which we call here florets. Fig 1 panel **A** visualizes the two different parts of the axonal arbor.

Characterization of florets based on 3D reconstructions of the axon happens in two steps: First, a mean-shift algorithm developed by Binzegger et al. [10] is used to find bouton-dense patches, which are candidate regions for florets. This process is repeated until all such clusters are identified. All axonal structures inside these clusters whose initial segment has come from side-branching are then defined as florets. A side-branch is defined as a branch that grows approximately perpendicular to the original direction of the axon, thereby distinguishing it from other bifurcations, which exhibit smaller branching angles between 20-80° [4, 38].

### Quantitative global and individual analysis of florets

Various indicators have been proposed for the characterization of neurite morphologies [9, 21, 29, 39]. We choose 2D indicators based on properties of the entire dataset (global indicators) as well as 2D indicators quantifying properties of individual florets (individual indicators). This allows us to account for overall governing growth rules as well as for individual floret morphology.

#### Global segment-length distribution

**Fig 2:**
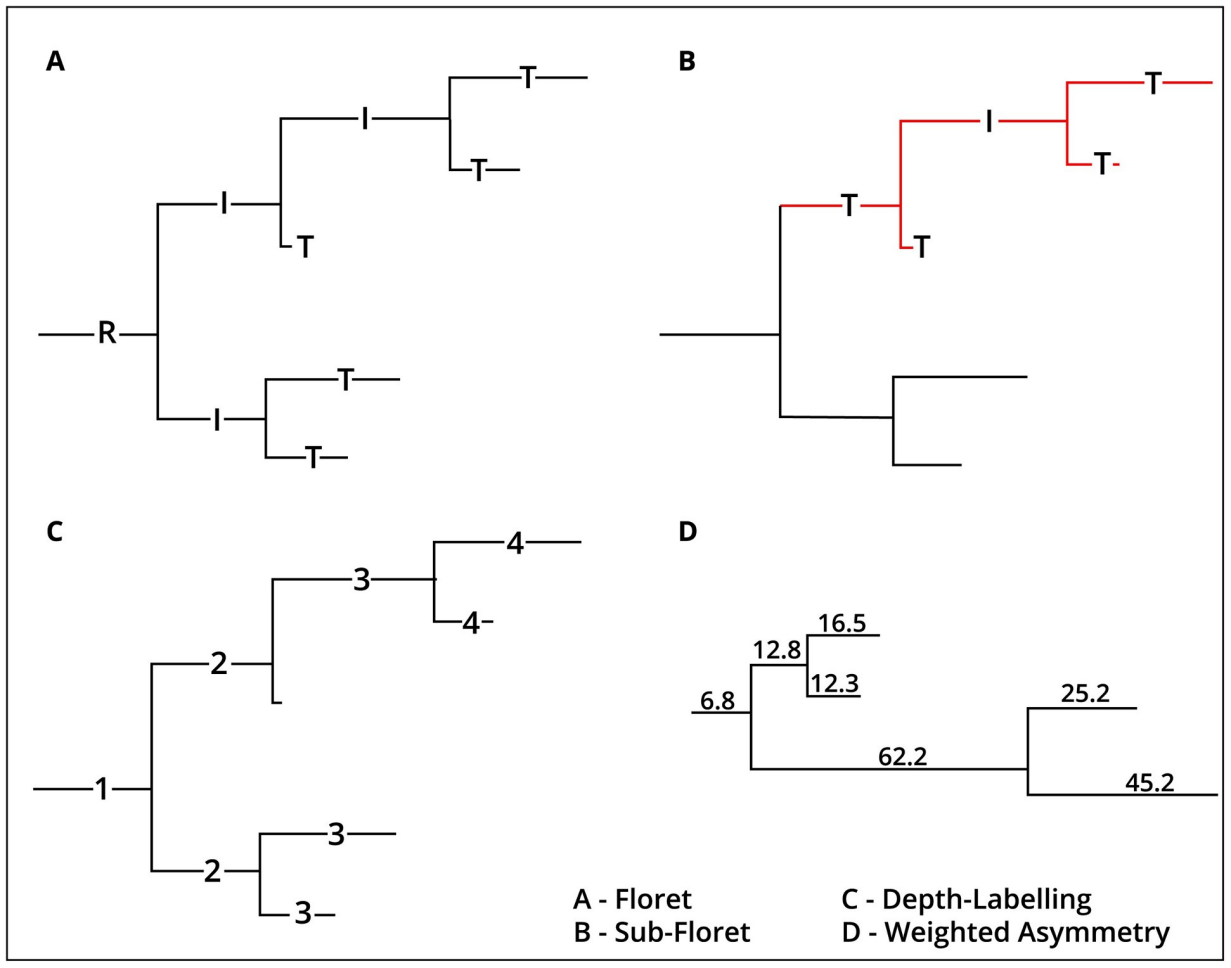
Visualization of axonal arbors with dendrograms. The indicators employed for evaluation of florets can be fully represented by dendrograms. (A) The dendrogram of a floret together with the different types of segments: Terminal segments (”T”), the root segment (“R”) and the internal segments (“I”). (B) An exemplary sub-floret is shown in red. (C) Each segment labeled by its depth. While the root segment has depth one, each subsequent bifurcation increments the depth by one unit. (D) For this floret from the dataset, the number of segments are identical for left and right sub-florets, the respective lengths, however, differ. Accordingly, the floret is perfectly symmetrical according to Van Pelt’s asymmetry index (asymmetry index = 0), but has an asymmetry of 0.41 according to the length-weighted asymmetry index.

Segment-lengths can be fully captured by 2D representations of growth morphologies called dendrograms. Dendrograms plot the segment-lengths along the x-axis while the branching topology is preserved (Fig 2). We distinguish here between the root segment, the internal segments, and terminal segments at the tips of florets. As florets are binary trees, each bifurcation leads to exactly two new segments. While the root segment has depth one, each subsequent bifurcation increments the depth by one unit. For an estimation of the global segment-length distribution first a histogram of all 2059 segments from the dataset is constructed. Then a maximum-likelihood fit is used to calculate the best fitting distribution to the histogram, where distributions are ranked by a Bayesian information criterion [40].

#### Length-weighted asymmetry: A novel quantification for neurite morphology

We introduce here a novel asymmetry index to quantify the asymmetry of neurites, called the length-weighted asymmetry index. It combines topological and metrical criteria, with topology understood as the connectivity pattern of the segments [41]. This index is an extension of a classical asymmetry index for tree-structures originating in the work of Van Pelt et al. [29] (and independently by Colless [28] in the field of phylogenetics) and extensively used in studies of neurite branching patterns [29]. Van Pelt’s index takes only the number of segments into account, independent from differences in segment lengths.

The length-weighted asymmetry index *A*_*w*_ employs the length of segments to weigh the contribution of each subtree, while being equivalent to Van Pelt’s index for binary trees with identical segment lengths. Let *F* be a floret with *n* segments *s*_*i*_, *i* ∈ *I* = {1, …, *n*}. Every segment *s*_*i*_ defines exactly one sub-floret *F*(*s*_*i*_) containing *s*_*i*_ as its root segment and all its descendants. Since every segment bifurcates into exactly two new segments, *F* has exactly $\frac{n-1}{2}$ non-terminal sub-florets. For every such sub-floret we define $s_{i_{l}}$ and $s_{i_{r}}$ as the left and right sub-floret originating from *s*_*i*_. Furthermore, for every sub-floret *G* of *F* we define |*G*| as the number of segments in the sub-floret, *t*(*G*) as the number of terminal segments in *G*, and $w\left(G\right)=\frac{1}{\left|G\right|}\sum_{s\in G}∥s∥$ as the mean segment length in *G*. We can now define the length-weighted asymmetry index *A*_*pw*_ for a sub-floret *F*(*s*_*i*_) as

$$A_{pw}\left(F\left(s_{i}\right)\right)=\{\begin{array}{cc} 0 & \text{if}\;t\left(F\left(s_{r}\right)\right)+t\left(F\left(s_{l}\right)\right)\leq 2\\ \frac{2|w\left(F\left(s_{r}\right)\right)t\left(F\left(s_{r}\right)\right)-w\left(F\left(s_{l}\right)\right)t\left(F\left(s_{l}\right)\right)|}{\left(t\left(F\left(s_{r}\right)\right)+t\left(F\left(s_{l}\right)\right)-2\right)\left(w\left(F\left(s_{r}\right)\right)+w\left(F\left(s_{l}\right)\right)\right)}\text{,} & \text{otherwise} \end{array}$$ (1)

Here, we compare the number of segments in all left and right subflorets, but weigh this number by the length of the respective segments. Finally, this value is normalized to lie in the unit interval [0, 1], as is the case for Van Pelt’s index. Similar to the index used by Van Pelt, the length-weighted asymmetry index of an entire floret *F* is computed as the mean of its non-trivial sub-florets’ asymmetry, summing over all sub-florets of *F*:

$$A_{w}\left(F\right)=\frac{2}{n-1}\sum A_{pw}\left(F\left(s\right)\right)$$ (2)

For both length-weighted and Van Pelt’s asymmetry index a value of zero corresponds to a fully symmetrical and a value of 1 to a completely asymmetrical floret. Fig 2 shows a floret from the dataset, which is fully symmetrical according to Van Pelt’s but not according to the length-weighted asymmetry index.

As the length-weighted asymmetry index proposed here takes metric as well as topological properties into account, it can also serve as a measure for quantifying the morphological diversity of florets (S1 Fig).

#### Further individual quantification criteria for florets

In addition to the length-weighted asymmetry index and the segment-length distribution, a number of other indicators, not used for optimization of the parameters of the floret-generator, are employed for the evaluation of generated florets (Table 1). A good match between biological and generated florets in terms of these criteria can hence serve as a further validation of the floret-generator. These additional indicators all quantify individual floret morphology based on concepts visualized in Fig 2. To compare generated and biological florets, a distribution of these indicators over the entire dataset is calculated.

**Table 1:** Evaluation criteria for individual generated florets.

The distributions of mean and standard deviation of segment lengths.	These measures capture the variability of segment-lengths between florets.
The distribution of mean and standard deviation of the logarithm of the segment-lengths.	Similar as above, now these measures allow for a quantification of the variability of the log of segment-lengths.
The distribution of number of segments.	This indicator reflects floret-size and branching occurence during the growth process.
The distribution of asymmetry values.	As length-weighted asymmetry is used to optimize the floret-generator, Van Pelt’s asymmetry index is employed for an evaluation of the individual results.
The distribution of floret depth.	Average depth is a topological indicator that reflects branching probabilities underlying the growth process.
The distribution of maximal depths.	Similarly as the indicator above, the maximal floret depth reflects underlying branching probabilities.

The criteria capture metric (segment-length) and topological (asymmetry, depth) properties, as well as floret size (number of segments). These criteria are not employed for the optimization of the floret-generator.

### Optimization of the floret-generator model

The parameters of the floret-generator model are optimized with a genetic algorithm (GA, for implementation details see Section “Parameter optimization with the genetic algorithm”.). In particular, we employ a GA to minimize the divergence of segment-length and length-weighted asymmetry distributions between biological and generated data to match global as well as individual statistics of the florets. This gives rise to the following objective function:

$$\alpha DM\left(D\left(L_{B}\right)∥D\left(L_{G}\right)\right)+\left(1-\alpha \right)DM\left(D\left(A_{B}\right)∥D\left(A_{G}\right)\right)$$ (3)

where $DM(D\left(L_{B}\right)∥D\left(L_{G}\right))$ denotes the divergence measurement (*DM*) between the distribution of segment-lengths of the biological dataset $D(L_{B})$ and of the generated dataset $D(L_{G})$. $DM(D\left(A_{B}\right)∥D\left(A_{G}\right))$ is the divergence between the distribution of length-weighted asymmetry indices of the biological dataset $D(A_{B})$ and of the generated dataset $D(A_{G})$. *α* is a scaling factor which was determined empirically (Section ‘Parameter optimization with the genetic algorithm’). As divergence measure the symmetric Jensen-Shannon (JS) divergence is employed [42]. Its symmetry ensures that an optimal trade-off for fitting peak as well as tail of the distribution is found.

### Statistical testing for equality of distributions

Tests for normality were performed by a Shapiro-Wilk test [43].

The two-sample Kolmogorov-Smirnov test implemented in MATLAB was applied to compare equality of distributions [44]. In particular, this test was employed for the comparison of biological and generated global segment-length and individual-floret distributions, and for the comparison of the same distributions from the MATLAB and the Cx3D implementations.

All tests were performed at the 5% significance level.

### Algorithmic encoding of the floret development process

Our model comprises the basic processes of growth, guidance, bifurcation and retraction [45, 46]. The dynamics are invoked by a module that represents the growth cone, a specialized structure at the tip of the neurite. Hence, the model is based solely on principles of self-construction [4, 11, 12, 25].

The growth and retraction lengths *l*_*growth*_ and *l*_*retract*_ are uniformly sampled from a gamma distribution, and if the retracted length is longer than the grown length, the respective branch is removed. The floret-generator model allows for the generation of varying segment-lengths within the same floret.

The floret-generator makes use of an abstract resource parameter to control the halting of the algorithm, which represents the tubulin provided to the distal end of the neurite [47, 48]. In particular, each floret is assigned an initial resource budget r, sampled from a gamma distribution. Each growth step requires one unit of resource, which is subtracted from the initial budget after the elongation of the growing segment. Retraction, in contrast, does not alter the resource budget.

After a branching process, we assume that both daughter growth cones follow the same rules as the parent growth cone [4, 49], while in possession of their own amount of resource. The distribution of resource to the new growth cones happens according to a random sample from [b,1], where b denotes the bias parameter. As the bias parameter is optimized in the interval [0.5, 1], the resource distribution will almost always be asymmetrical, in agreement with the asymmetry found in the dataset. Given this amount of resource, the growth cones are independent from one another, and hence independent of the overall topology of the grown floret.

The model employs a growth offset: each floret grows an initial minimum length os, which can be understood as the minimal length given by basic building blocks of the neurite such as microtubules. The offset can furthermore account for the characteristic lack of short segments in the dataset (see also [21] for a similar offset element in dendrite growth models).

### Parameter optimization with the genetic algorithm

Mathworks’ built-in GA from the Global Optimization Toolbox, Version 3.4.1., was used to optimize the parameters of the floret-generator [50]. The optimization is performed according to the following steps: In a first step, the GA randomly generates initial solutions. In subsequent steps, ten percent of solutions with the best fitness values (”elites”) are guaranteed to be passed on to the next generation, while additional solutions for the next generation are created through crossover and mutation. The algorithm halts if a maximum number of generations, here set to ten times the number of parameters, is reached or if the weighted average change over 50 generations falls below 10^−6^. In particular, the Jensen-Shannon divergence was calculated between normalized histograms of the biological and the generated data for the objective function presented in Eq 3. Information on relevant settings of the GA as well as on values for the initial populations can be found in S1 Text and in S1 and S2 Tables, respectively. Histograms were constructed with MATLAB’s built-in histogram method [51]. *α* has been selected based on preliminary assessments of the log-normal fit and without taking the evaluation criteria into account: a value of 0.9 yields a good fit to the segment-length distribution while also generating the appropriate range of floret complexities. Further implementation details and requirements to run the accompanying software can be found in S1 Text.

## Results

We present the global and individual statistics of the biological dataset, and compare these to the statistics of the generated florets. We begin by discussing the results of the Galton-Watson model for the dataset, and then proceed to the floret-generator model. After presenting the statistics of the floret-generator model, we present the Cx3D implementation, where biophysical interactions with the environment are incorporated in the axonal growth process.

### Global segment-length distribution of the floret dataset

**Fig 3:**
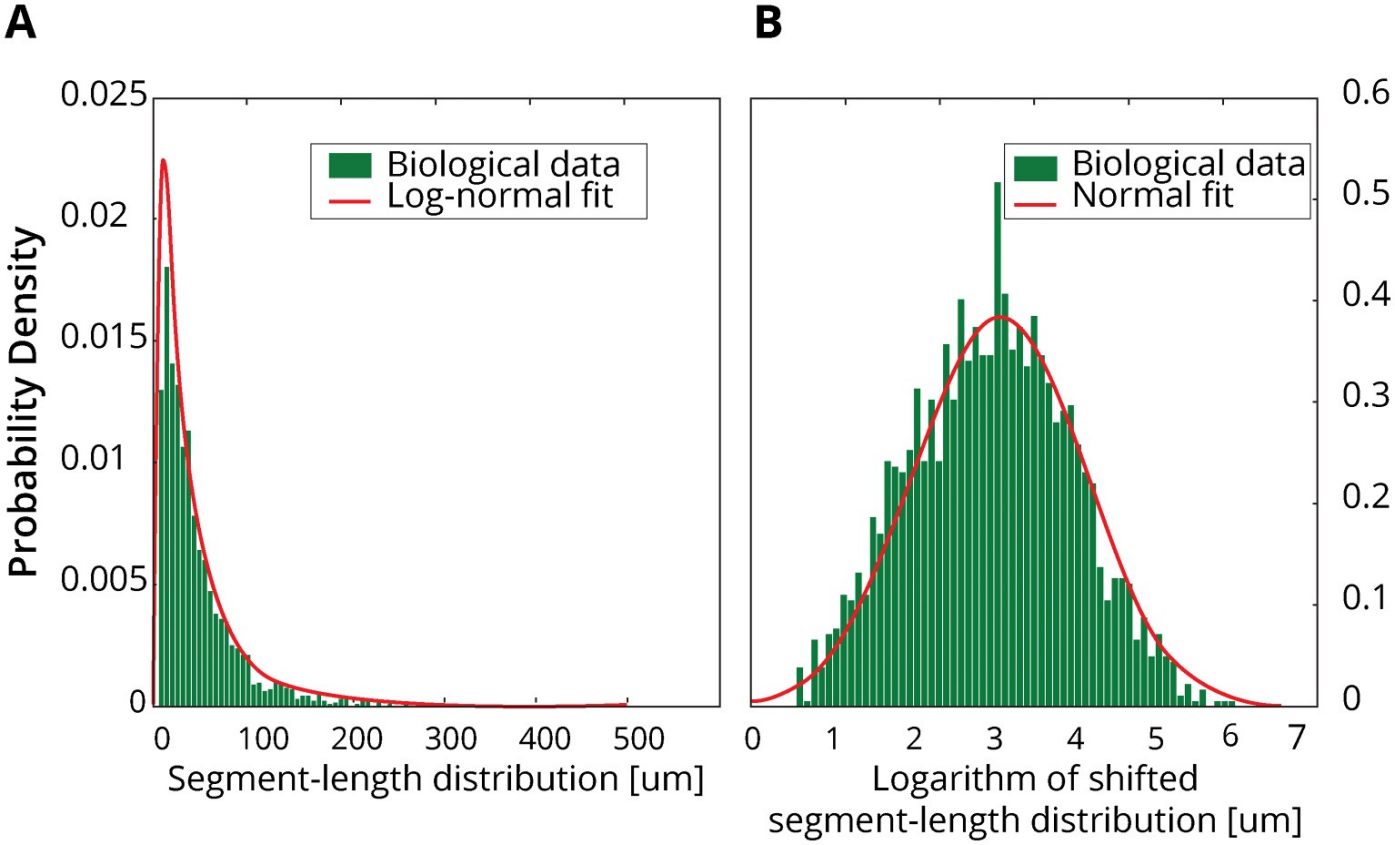
Segment-length distribution of the biological data. (A) Histogram of all 2059 segment-lengths of the dataset and best maximum-likelihood fit. The best fit is achieved by a log-normal distribution. (B) Histogram of the logarithm of the shifted segment-length distribution. The histogram displays now a skewness of only −0.0636. The Shapiro-Wilk test [43] now fails to reject the null-hypothesis of normality (W_2059_ = 0.99, p > 0.16), suggesting that the data follows a shifted log-normal distribution.

**Fig 4:**
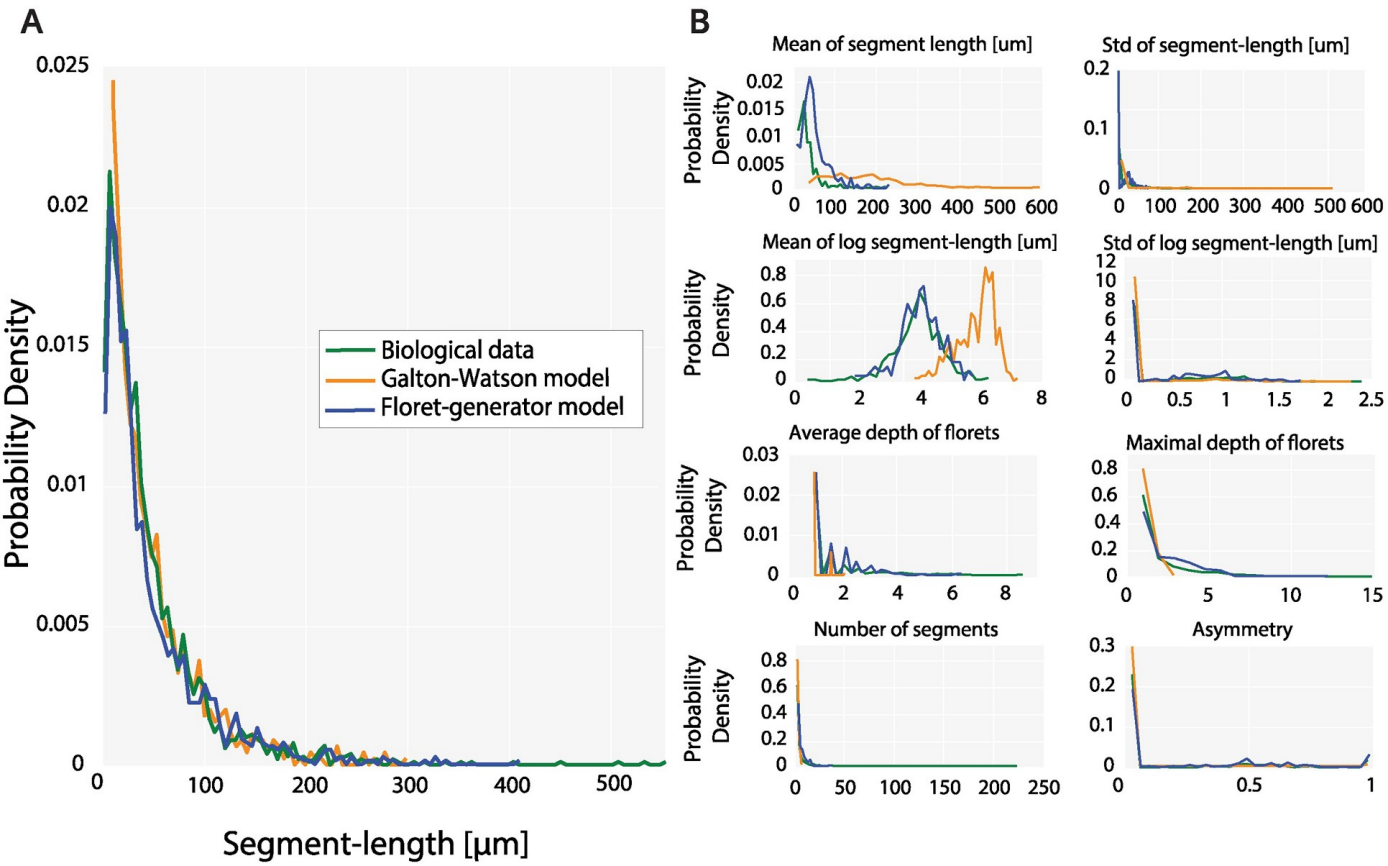
Comparison of generated and biological segment-length distributions and individual statistics. (A) The best generated run from the Galton-Watson model displays a rather high Jensen-Shannon divergence (0.026). This can be explained by the model’s failure to recreate the lack of short segments, and the tail of the biological data. The null-hypothesis of equality is rejected by a two-sample Kolmogorov-Smirnov test (D_2059,1724_ = 0.0621, p = 0.0083). The divergence between the segment-length distribution of the floret-generator and the biological data is lower (Jensen-Shannon divergence of 0.011), indicating a good overall fit. As can be seen, the generated segment-length distribution matches peak as well as tail of the biological segment-length distribution. The equality of these two distributions is underlined by a two-sample Kolmogorov-Smirnov test (D_2059,1678_ = 0.0302, p > 0.3). (B) Comparison of the generated and biological individual statistics. The biological florets display a high variability with respect to asymmetry and segment-lengths. The lack of the short segment cannot be recreated by the Galton-Watson distribution. Accordingly, the generated segment-lengths do not match the biological data as adequately as the floret-generator is capable of. Indeed, with the exception of the asymmetry distribution, the two-sample Kolmogorov-Smirnov rejects the null-hypothesis of equality for all individual distributions (D_426,500_ = 0.1182, p < 0.002). The results of the floret-generator, however, capture the fraction of trivial florets which are completely symmetric and have depth one. Concurrently, the generated florets are also reproducing the entire ranges of the indicators.

The global segment-length distribution of the biological data (Figs 3 and 4 panel **A**), containing a total of 2059 segments, displays a long-tailed unimodal distribution with a visible lack of short segments. This distribution is found separately for the intermediate and the terminal segments as well (S2 Fig). Such a distribution has previously also been observed for dendritic data [21, 23, 52]. The best fit is achieved by a shifted log-normal distribution with a shift parameter *γ* = −2.9157, and parameters *μ* = 3.52 and *σ* = 1.03, estimated with MATLAB’s fminsearch [53]. Without the shift, the logarithm of the segment-length distribution displays a skewness of −0.2726. Indeed, the Shapiro-Wilk test for normality of the logarithm of only the shifted segment-distribution fails to reject the null-hypothesis of normality (W_2059_ = 0.99, p > 0.16).

### Individual statistics of the floret dataset

Key statistical properties and the individual quantification criteria of the biological dataset are presented in Table 2 and Fig 4B respectively. 260 out of the 426 florets of the dataset are trivial florets. These florets have only one segment, and accordingly are fully symmetrical and have a depth of 1. In contrast, non-trivial florets of the dataset have on average 10.84 branches and an asymmetry of 0.39, with an outlier floret consisting of 232 branches. Segments with lengths below 1 micron were discarded due to constraints in measurement precision, and one outlier segment with a length of over 900 microns was discarded as it was found to be clearly outside of the range of the remaining lengths (from 1 to around 540 microns). The morphological variability of the florets is captured by the distribution of standard deviations of segment-lengths and log segment-lengths, which display that a considerable fraction of florets contain segments of highly variable lengths.

**Table 2:** Statistical properties of the floret dataset from cat thalamic axons and the three generated datasets.

	Biological data	MATLAB	Cx3D	Galton-Watson
Mean segment-lengths	47.5 *μ*m	52.57 *μ*m	53.1 *μ*m	312.5 *μ*m
Mean segment-lengths NT	48.89 *μ*m	45.49 *μ*m	45.8 *μ*m	134.58 *μ*m
Mean segment-lengths T	50.55 *μ*m	68.2 *μ*m	64.1 *μ*m	355.55 *μ*m
Mean SD segment-lengths	15.99 *μ*m	23.3 *μ*m	23.3 *μ*m	19.06 *μ*m
Mean SD segment-lengths NT	41.04 *μ*m	36.1 *μ*m	36.2 *μ*m	97.84 *μ*m
Mean depth	1.69	1.77	1.78	1.13
Mean depth NT	2.79	2.2	2.2	1.67
Mean asymmetry	0.15	0.24	0.21	0.01
Mean asymmetry NT	0.39	0.35	0.35	0.02

Individual statistics of the biological data can be found in the leftmost column. The mean asymmetry of the biological data equals 0.15, a rather low value which can be explained by the large number of trivial florets present in the dataset. The mean asymmetry of the non-trivial florets equals in contrast 0.39. The variations of the segment-lengths additionally underline the morphological variability of the biological data. Columns 2 and 3 reveal that the floret-generator, both in MATLAB and Cx3D, matches the biological data best. The Galton-Watson model diverges markedly from the statistics of the dataset with regard to the generated segment-lengths. This can be ascribed to its failure of reproducing peak and tail of the segment-length distribution of the biological data. This measure can be matched well by both floret-generator and Galton-Watson model. SD: Standard deviation; NT: Non-trivial floret; T: Trivial floret. Where not indicated otherwise, the criterion is calculated over the entire dataset.

### Evaluation of the Galton-Watson model

Here, we fit the Galton-Watson model to the biological dataset, and compare the generated and biological global and individual statistics. The Galton-Watson model allows at each step for three possible actions: Bifurcation, growth or halting. The respective probabilities p_*b*_, p_*gr*_, p_*st*_, and the elongation length at each step, remain constant during the entire growth process and generate an exponential segment-length distribution [9, 18].

In accordance with the work of Binzegger et al. [9] we chose here a segment-length equal to 1. As growth and branching probabilities sum up to 1, we optimize with the GA only p_*b*_ and p_*gr*_ with respect to the objective function defined in Eq 3, and create 500 artificial florets. In line with Binzegger et al. [9], we use the condition 2*p*_*b*_ + *p*_*gr*_ < 1 to prevent infinite growth [18].

The optimized Galton-Watson model has a growth probability p_*gr*_ of 0.98 and a branching probability of p_*b*_ of 0.0031, comparable to the results of Binzegger et al. [9]. As the model generates an exponential segment-length distribution (Fig 4A), it is not capable of recreating the lack of small segment-lengths as observed for the biological data. This discrepancy between artificially generated florets from the Galton-Watson model and the biological data is also apparent in the individual statistics (Table 2) where the Galton-Watson model fails at recreating branches with accurate lengths and morphological properties. With the exception of the asymmetry distribution, equality of generated and biological distributions is rejected by a two-sample Kolmogorov-Smirnov test for global (p = 0.0083) as well as individual statistics (p < 0.002).

### The floret-generator model

**Fig 5:**
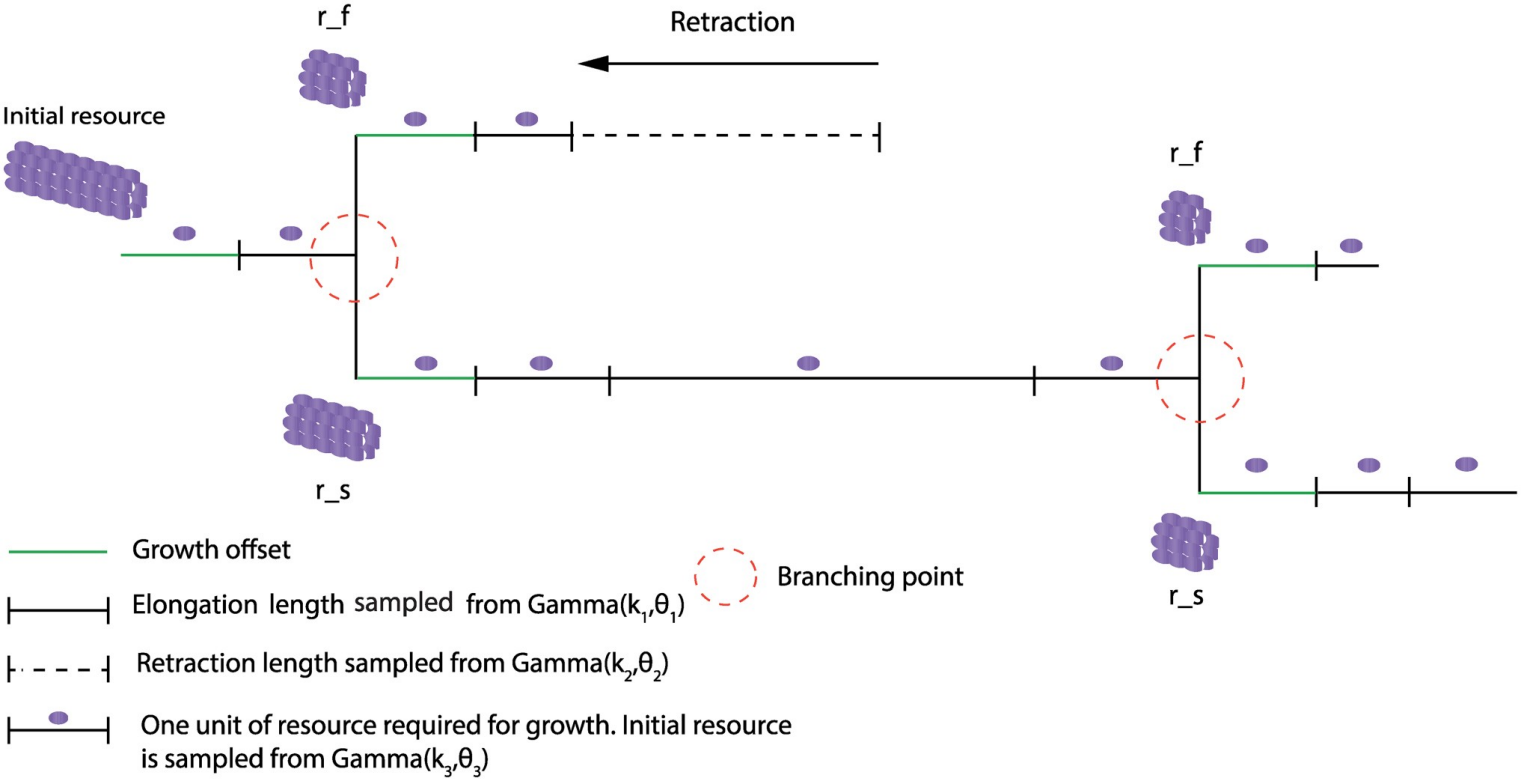
Components of the floret-generator algorithm. The floret-generator allows for growth, branching or retraction. Growth and retraction lengths are both sampled from respective gamma distributions. When a growth event occurs, an initial offset length is formed first. An initial resource budget is sampled from a Gamma distribution, and resource is reduced by one unit after each growth step. After branching, a bias parameter decides on the distribution of resource to the daughter segments. The parameters of the gamma distributions as well as all other parameters of the model are optimized by a genetic algorithm.

The observation that axonal ramifications in the thalamocortical target area display self-similarity [9] speaks in favor of a repeated realization of the same set of rules through the growth cones. This motivates us to model our floret-generator from the viewpoint of independent agents, which carry out the growth, bifurcation and retraction events according to the same set of rules and locally available information, as elaborated in Section “Algorithmic encoding of the floret development process”. The algorithm of the floret-generator is illustrated in pseudo-code in Box 1, its parameters are presented in Table 3. The different steps of the algorithm are presented schematically in Fig 5. All parameters of the model are optimized with a GA as described in Sections “Parameters and optimization of the floret-generator” and “Parameter optimization with the genetic algorithm”.

**Box 1. The algorithm of the floret-generator in pseudo-code**

1. Set *r* to the initially available amount of resource

2.  (a) Grow the initial segment with an offset length *os*

  (b) Reduce resources by *r* ← *r* − 1

while *r* ≥ 1

  3. Draw a uniform random number *p* ∈ [0, 1]

  4. If *p* ≤ *p*_growth_, perform *growth* step, otherwise go to Step 6:

  5. If next step is *growth*:

   (a) Elongate the current neurite by a length *l*_*growth*_

   (b) Reduce resources by *r* ← *r*−1

  6. If next step is not *growth*:

   (a) Draw a uniform random number *q* ∈ [0, 1]

   (b) If *q* < *p*_retract_ perform *retract* step, otherwise perform *bifurcation*:

   (c) If next step is *retract*:

    (1) Reduce length of current segment by *l*_*retract*_

    (2) If remaining length of the segment is < 1, remove it and terminate.

   (d) If next step is *bifurcation*:

    (1) Draw a uniform random number *z* ∈ [*b*, 1], where *b* is the bias

    (2) Distribute resources according to *z*:

     give 1 + (1 − *z*) ⋅ (*r* − 2) resources *r*_*f*_ to the first branch

     give 1+ *z* ⋅ (*r* − 2) resources *r*_*s*_ to the second branch

    (3) If resources for both new branches are >1

     continue at step 2 recursively for both branches,

     otherwise terminate.

  end if

end while

**Table 3:** Parameters of the floret-generator with optimized values.

	Parameter description	Range	Optimal Value
Growth parameters	Elongation lengths [*l*_growth_] are drawn from a gamma distribution with optimized shape and scale parameters.	[g_*Sh*_, g_*Sc*_]	[g_*Sh*_, g_*Sc*_]
		[0, 100]	1.26,21.18
Retraction parameters	Retraction lengths [*l*_retract_] are drawn from a gamma distribution with optimized shape and scale parameters.	[r_*Sh*_, r_*Sc*_]	[r_*Sh*_, r_*Sc*_]
		[0, 100]	1.69, 17.82
Resource parameters	For every floret, the amount of available resource *r* is drawn from a gamma distribution with optimized shape and scale parameters.	[rs_*Sh*_, rs_*Sc*_]	[rs_*Sh*_, rs_*Sc*_]
		[0, 20]	14.99,11.29
Growth and retraction probabilities	The probability of growth and retraction are both sampled uniformly from [0, 1].	[*p*_growth_, *p*_retract_]	[*p*_growth_, *p*_retract_]
		[0, 1]	0.11,0.58
The bias parameter	The bias parameter is optimized in the range of [0.5,1]. It determines the allocation of resource after bifurcation.	[b]	[b]
		[0.5,1]	0.63
The offset parameter	The offset parameter determines the initial length grown.	[os]	[os]
		[1, 2]	1.76

The parameters of the floret-generator are optimized by a genetic algorithm within their individual ranges. The genetic algorithm seeks an optimum over several iterations of its search process by minimizing the divergence of segment-length and length-weighted asymmetry distributions between biological and generated data. The right-most column summarizes the results of the optimization process. The optimized bias parameter is close to 0.6, indicating a rather equal distribution of resource after branching events.

#### Parameters and optimization of the floret-generator

The model parameters are presented in Table 3. The parameters of the floret-generator are optimized with the GA with respect to the objective function defined in Eq 3. These parameters are optimized by taking the entire biological dataset into account. During the growth process, however, each floret randomly samples from these optimized distributions.

#### Segment-length distribution of the generated floret dataset

The generated segment-length distribution based on 500 generated florets and the parameters optimized with the GA are presented in Fig 4A. As visible, the segment-length distribution matches the peak as well as the long tail of the segment-length distribution of the biological dataset. The generated distribution is also capable of accounting for the lack of short segments characteristic of the biological dataset. In summary, the generated segment-lengths cover the entire range of the original segment-lengths but also reflect the variability of these, hence reproducing the shifted log-normal distribution found in the original dataset. The good fit is underlined by the results of a two-sample Kolmogorov-Smirnov test for the equality of the distributions (D_2059,1678_ = 0.0302, p > 0.3). Repeated realizations of the floret-generator based on the same set of parameters underline this result (S3 Fig).

**Fig 6:**
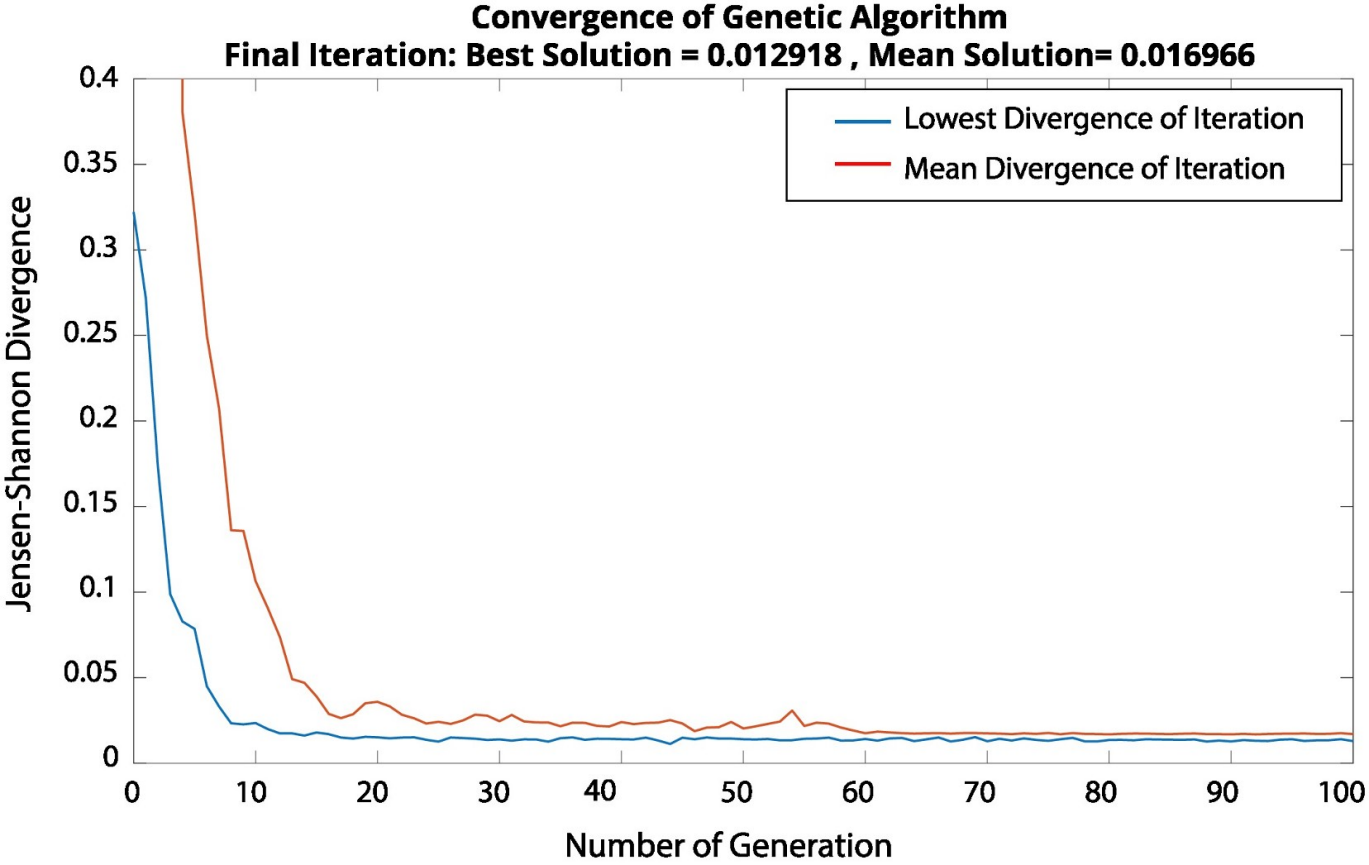
Convergence of the Genetic Algorithm. The genetic algorithm employed for optimization of the parameters of the floret-generator. Both the mean as well as the lowest Jensen-Shannon divergences associated with each generation of the genetic algorithm converge quickly to a stable value. The genetic algorithm halts if the maximal number of generations, by default set to ten times the number of parameters, is reached, or, if the weighted average change over 50 generations falls below 10^−6^.

The optimized parameters of the floret-generator are displayed in Table 3. Solutions with other sets of optimized parameters reveal that the results of the floret-generator are robust with regard to slight changes in the parameters (S4 and S5 Figs). Fig 6 shows the convergence of the GA for a representative run. Displayed are the best run of each generation, as well as the mean divergence value per generation.

The optimized bias parameter is close to 0.6, indicating a rather equal distribution of resource after branching events, in accordance with the rather low asymmetry of 0.15 of the biological dataset. The parameters of retraction indicate that large lengths are pruned by retraction. As parameters for the resource distribution are close to the upper bound of their optimization interval, this might indicate repeated cycles of growth and retraction until the variable morphology of the florets is obtained.

In fact, a model without retraction is not capable of replicating the lack of short segments found in the biological data, and hence fails to match the peak of the biological distribution (S6 Fig).

#### Individual statistics of the generated floret dataset

The individual statistics from the optimized floret-generator are shown in Fig 4B. As can be seen the generated florets capture the statistical properties of both trivial and non-trivial florets found in the dataset. This becomes apparent upon inspection of depth, segment number and asymmetry distributions, where the generated population captures the large fraction of symmetrical florets with only one segment and a depth of 1 and concurrently matches the distributions of the standard deviation of segment-lengths and log segment-lengths of the biological dataset. This demonstrates that the model can create highly variable florets, not only with globally matching statistics but also with similar individual morphologies as the biological arbors. With the exception of depth and standard deviation of segment length distributions, the Kolmogorov-Smirnov test fails to reject the null-hypothesis of equality for all individual statistics (p > 0.2).

### The floret-generator in Cx3D

Using the model parameters optimized in MATLAB, we port the floret-generator to Cx3D (“Cortex 3D”), a 3D environment for the simulation of neural development [30]. Cortical development in Cx3D is implemented in accordance with principles of cortical self-construction, where the computational building blocks are locally autonomous processes.

Assuming that growth cones follow chemotaxic cues [31–33], we model here the morphological asymmetry of florets as dependent on an abstract substance in the neurites’ extracellular matrix. In particular, we assume that resource allocation is now governed by the norm of the gradient of the substance: When bifurcating, the neurite senses and “extracts” the gradient of the surrounding substance for resource allocation. The concentration of the substance is modelled here as constant along the *x*- and *y*-axis, but linearly changing along the *z*-axis, the florets’ direction of growth. We now define the bias parameter, as introduced in Table 1, as the slope of the substance along the *z*-axis, whereby again 1 > *bias* > 0.5. As the gradient vector *v* of the substance equals (0, 0, *bias*), its norm is equal to the bias parameter, i.e. ∥*v*∥ = *bias*. Assuming that gradient detection through the growth cones is noisy [54], we match our previous usage of the bias parameter (Table 1) by adding noise to the bias parameter. In particular, we sample uniformly from the unit interval, and multiply the sample by (1 − ∥*v*∥).

In order to achieve growth into the direction of the gradient we allocate the larger fraction of resource to the segment enclosing a smaller angle with the gradient. In this manner, the asymmetrical growth of neurites is modelled through an interaction of the neurite with its extracellular environment.

**Fig 7:**
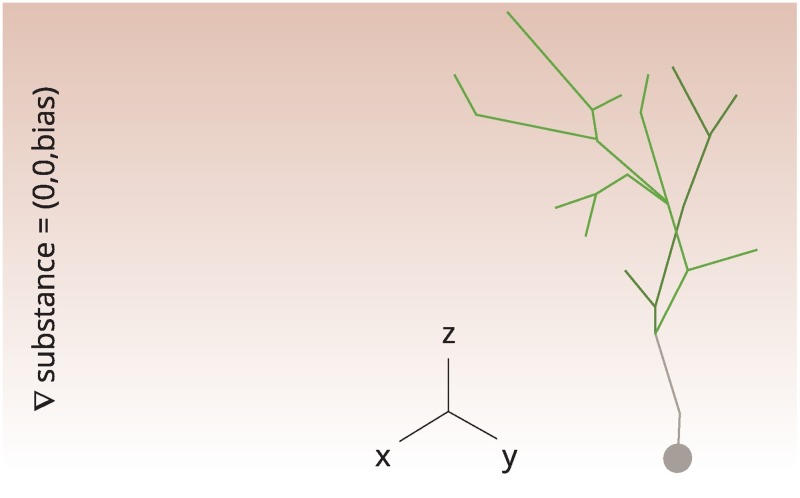
A 3-dimensional floret generated in Cx3D. An extracellular substance is shown in the background (red). The gradient of the substance changes linearly along the z-axis, with the norm of the gradient being interpreted as the bias parameter. After bifurcation, the norm of the gradient plus an additional noise component determine how resource is distributed to the two offspring growth cones.

As the optimization of the floret-generator is not taking the angles between segments into account, such angles are generated based on a random procedure. In particular, the two daughter branches re-orient themselves from the mother branch via a random rotation at each branching point. To this end, the daughter branches rotate by 30 degrees in the opposite direction from one another. This yields an angle of 60 degrees between them, while the rotation axis is drawn randomly. An exemplary Cx3D-generated floret is shown in Fig 7.

The floret-generator can be successfully ported into Cx3D, as the segment-length distributions and individual statistics generated in this environment correspond to the statistics resulting from the MATLAB implementation (S7 Fig). This successful translation of the floret-generator further underlines its agreement with biological principles of cortical self-construction.

## Discussion

”Patchy” regions of thalamic arborizations have been observed in various species, and these local ramifications are thought to preserve the spatial resolution of the associated receptive fields and enable dense synaptic connections for the ocular dominance columns [1–3]. Although the axonal tree reveals a particular complex morphology, the structural regularities of the neocortex [5] suggest a minimal set of growth rules which are carried out locally in interaction with their environment. Our results demonstrate indeed that the morphologies of axonal ramifications of cat thalamic afferents can be recreated with remarkable accuracy from the repeated realization of only few simple growth rules. Based on a small set of locally autonomous growth processes in accordance with developmental principles of the brain [4, 5, 12, 13], the model is capable of matching the global shifted log-normal distribution of the segment-lengths, a stark improvement to the exponential segment-length distribution generated by the Galton-Watson model [9]. As the Galton-Watson model fails to replicate the fraction of small segments, the statistics generated by this model are also much less in accordance with the morphologies of individual biological arbors.

The good fit achieved to the global statistics of the biological data can be attributed to several properties of the floret-generator. The large expected value of resource together with a low branching probability—which implies that numerous steps of growth can occur before the next branching point—can be attributed to matching the tail of the global segment-length distribution. Occurrences of retraction can in addition prune some of the grown segments to smaller lengths. This leads to artificial florets with metrically highly variable segments, as present in the original dataset. Taken together this explains the capability of the floret-generator to match the peaks as well as the tails of the log-normal distribution.

A future extension of our work lies in the inclusion of electrical signaling activity [12], where activity emerges based on developmental self-construction of the local circuitry. While the electrical activity of neurons is the topic of intense studies, it is rather rarely assessed in the context of neurite development. However, neurite growth, branching and retraction during development are strongly influenced by electrical activity [55–57], and in turn determine the signaling properties of neuronal circuits [58, 59]. Neurite outgrowth based on local principles has been recently studied within the self-construction of local cortical circuits, where the self-calibrated activity gives rise to winner-take-all networks with computational properties such as signal restoration and amplification [12]. Understanding the rules underlying neurite branching patterns is hence crucial for our understanding of the local neocortical circuitry, thought to lie at the heart of cortical computation [5–7].

By embracing a mechanistic modelling approach in line with the principles of cortical self-construction [3, 9, 12, 60], our model furthermore provides the basis for empirically testable predictions about the axonal growth process. Our model predicts, for instance, frequent occurrences of retraction while concurrently retraction lengths are similar to elongation lengths. While retraction of dorsal LGN projections is known to be crucial for the formation of ocular dominance columns [45], the results of the presented model allow for empirical tests of these particular properties.

With the goal to provide a biophysical interpretation for statistical observations, the floret-generator was ported into the simulation environment of Cx3D, where the bias parameter was understood as the gradient of an extracellular cue, linearly changing along the axis of growth. Again, this implementation did not assume any kind of global coordination between the growth cones as opposed to previous work in the literature [19, 61]. After bifurcation, the larger amount of resource was allocated to the branch enclosing a smaller angle with the extracellular gradient plus noise, reflecting the emiprical observations about the role of chemotaxic cues for axonal path finding [31–33]. The asymmetric allocation of resource allows the model also to generate topologically variable structures. Despite the 3D environment of Cx3D, however, we had restricted the optimization of the model to two-dimensional properties of the biological florets. A future inclusion of angles and other 3D properties of the axonal morphologies might lead to further conclusions about the underlying growth principles. It is known, for instance, that axons do not fully align with the direction of the relevant gradient [62, 63]. Our model provides a mechanistic framework for the optimization of angles where such additional properties can be integrated.

Conditioned on the available resource, the presented floret-generator model falls into the category of Bellman-Harris processes [64], a generalization of the Galton-Watson branching process [18, 65]. While the Galton-Watson process has been employed in neuroscientific studies [9, 24, 66–68], our results suggest that modelling neurite outgrowth using the Bellman-Harris process constitutes a valuable addition. With regard to neurite modelling, the Bellman-Harris process can be understood as a growth process where the elongation lengths are generated from an arbitrary distribution, and where they are independent of one another. Evidently, modelling neurite morphology according to the Bellman-Harris process allows for bigger flexibility than the Galton-Watson process, which assumes constant elongation lengths. Subsequent analysis of Bellman-Harris processes with respect to neurite growth processes might allow for further valuable deductions with regard to these growth processes.

The simplicity of the proposed model, as well as its capability to match the log-normal segment-length distribution based on various parameter sets, promise a generalization to other neurite morphologies. Accordingly, future studies should assess the application of the model to such other experimentally collected datasets. Furthermore, the model could be integrated into broader simulations of cortical development [4, 11, 12]. As X- and Y-projections have functionally distinct characteristics, a separate assessment of the growth model for the two categories could furthermore allow for distinct analysis and predictions for florets associated with each cell type. The granularity of simulated behaviours in our floret-generator model is such that multiple growth or retraction events can occur before the branching event. Within the Cx3D framework, individual events such as multiple growth or retraction events occurring before a branching event, can be modelled explicitly by respective ‘modules’ [30]. This approach allows for a future validation of the proposed model based on such small movements from experimental observations. Hence, this theoretical model can be put in closer comparison with experimental data than if the granularity was coarser. As previous analysis have revealed a right-skewed, unimodal distribution for dendrite segment-lengths [21, 23, 52] similar to the segment-length distribution of the here assessed axonal data, it is worthwhile to apply the presented model to dendritic data. Differences in parameters of the model could subsequently allow to further dissociate between axonal and dendritic growth rules.

In summary, the proposed model that the elaborate thalamic arborizations in cat area 17 can be accurately replicated with only a small set of locally carried out rules, in line with previous work supporting the hypothesis of the uniformity of cortical structure [5–7, 9–12]. The mechanistic framework offered here allows for its further extension by the integration of additional environmental components, supporting future empirical assessment of neurite growth characteristics.

## Supporting information

S1 Text Implementation details and background on supplementary figures. Details on the implementation of the presented floret-generator algorithm and requirements for running the available code are provided here. In addition, background for all results presented in supplementary figures is provided.
(PDF)

S1 Fig Weighted asymmetry as a quantification of morphological complexity. (A) Dendrograms of the florets from the biological dataset visualize the morphological diversity of the florets. These range from structures with only a single branch (top left) to morphologies with many branches of different lengths and topological orderings. (B) Weighted asymmetry values are plotted for the dendrograms presented in panel A, from left to right. Higher morphological complexity is reflected by an increased weighted asymmetry value, as this quantification considers both the length of the segments as well as their placement within the arborization pattern.
(TIF)

S2 Fig Segment-length distribution of intermediate and terminal segments. (A) The segment-length distributions of the terminal and (B) intermediate segments. Although the distributions are not entirely identical, they are in all cases unimodal, right-skewed and long-tailed. Additionally, the mean segment-lengths of intermediate and terminal segment are with 47.14 microns and 47.14 microns respectively remarkably similar. In addition, a two sample ks-test can not reject the equality of the distributions (p > 0.4).
(TIF)

S3 Fig Results of the floret-generator with confidence interval. A 90% confidence interval is constructed from 100 realizations of the floret-generator with parameters optimized by the genetic algorithm (Table 3). (A) As visible, the segment-length distribution from the different runs replicates the unimodal distribution of the biological data closely. (B) We observe also a good fit of the generated data to the individual statistics of the biological florets, based on measures not employed for optimization.
(TIF)

S4 Fig Results of the floret-generator with alternative parameter sets. We construct a 90% confidence interval from the generated data based on a first alternative sets of optimized parameters and 100 realizations of the floret-generator. (A) Both the segment-length distribution, (B) as well as the individual statistics of the biological data, can be matched well based on the two parameter sets. We hence conclude that the good results obtained by the floret-generator are robust with respect to different solutions of the optimization process.
(TIF)

S5 Fig Results of the floret-generator with alternative parameter sets. Here, we construct a 90% confidence interval from the generated data based on a second alternative sets of optimized parameters and 100 realizations of the floret-generator. (A) Both the segment-length distribution, (B) as well as the individual statistics of the biological data, can be matched well based on the two parameter sets. We hence conclude that the good results obtained by the floret-generator are robust with respect to different solutions of the optimization process.
(TIF)

S6 Fig Results of the floret-generator without retraction. Panel (A) and (B) display the generated segment-length distribution of the floret-generator without retraction, based on the parameters optimized by the genetic algorithm. (A) The confidence interval based on 100 instantiations of the optimal parameters. The fit to the peak of the biological data is not optimal as the characteristic lack of small segments observed in the biological data can not be matched by this model. (B) The individual statistics of the floret-generator model without retraction, where the generated florets have typically only few segments.
(TIF)

S7 Fig Comparison of statistics of the MATLAB and the Cx3D implementations. (A) Comparison of the global segment-length distribution from the floret-generator implemented in MATLAB and in the physical simulation environment provided by Cx3D. The distributions are based on 500 generated florets in each implementation. Although the distribution of resource is modelled in Cx3D as an interactive process influenced by extracellular gradients, the global statistics generated by both implementations proves to be equivalent. (B) Comparison of the individual statistics for the floret-generator implemented in MATLAB and in Cx3D. The individual statistics are calculated based on 500 generated florets in each implementation. The individual statistics show a close match, confirmed by a two-sample Kolmogorov-Smirnov test for both global and individual statistics (p > 0.8). The slight divergence in the results can be attributed to the stochasticity of the floret-generator.
(EPS)

S1 Table Parameters of the Genetic Algorithm. Parameters of the genetic algorithm employed for the optimization of the floret-generator. All other parameters are set to default values of the genetic algorithm from MATLAB’s Global Optimization Toolbox. PopulationSize: The number of solutions (also called ‘individuals’) in each generation. EliteCount: The fraction of the top-solutions passed on to the next generation. CreationFcn: The function creating the initial population for the genetic algorithm. SelectionFcn: The function which determines how the genetic algorithm chooses ‘parents’ for the next generation. ‘Parents’ are two solutions from which a new solution is generated. CrossoverFcn: The function specifies how the genetic algorithm combines two solutions to form a new solution for the next generation. MutationFcn: The function specifies how the genetic algorithm makes small random changes a solution in the population to create a new solution for the next generation.
(PDF)

S2 Table Initial parameter values of the Genetic Algorithm. The initial parameter values of the floret-generator of an initial population of the genetic algorithm. Parameter values are selected for the initial populations probabilistically within their individual ranges (for the ranges see main text Table 3). Over the following iterations, the genetic algorithm seeks an optimum of its search process by minimizing the divergence of segment-length and length-weighted asymmetry distributions between biological and generated data.
(PDF)

S3 Table Optimized parameters of the floret-generator. Two alternative parameter sets of the floret-generator model optimized by the genetic algorithm. As visible, the parameters are remarkably similar to the best solution presented in Table 3.
(PDF)

S4 Table Optimized parameters of the floret-generator without retraction. The parameters of the floret-generator model without retraction as optimized by the genetic algorithm. Shape and scale parameters of the gamma distribution for growth are considerably higher as compared with the same parameters of the model with retraction. The growth probability and the parameters of the resource distribution are in contrast significantly smaller.
(PDF)

S1 Data Underlying data for biological and generated florets. The segment-lengths as well as all data underlying the individual statistics of florets are provided for the biological data, for the floret generator models with and without retraction, and for the implementation of the floret-generator in Cx3D.
(XLSX)
