# Supplementary material for: A generative growth model for thalamocortical axonal branching in primary visual cortex
Source: PLoS Comput Biol. 2020 Feb 13;16(2):e1007315. doi: 10.1371/journal.pcbi.1007315 (PMC7018004; doi:10.1371/journal.pcbi.1007315)
Supplement: S1 Text — Details on the implementation of the presented floret-generator algorithm and requirements for running the available code are provided here. In addition, background for all results presented in supplementary figures is provided. (PDF) [file pcbi.1007315.s001.pdf]

Supporting Information

S1 text: Supporting Information

Implementation details and requirements

The biological floret data for the here presented study is provided in an XML-format, where each floret is described by a set of points in three-dimensional euclidean space. These points represent the end- and starting points of segments, whose lengths are in addition explicitly provided in the XML file. Both the XML file and the code for the floret generator with instructions are provided online under https://www.research-collection.ethz.ch/handle/20.500.11850/313373 and with regular updates under https://github.com/Pegahka/GenerativeAxonModel

A tutorial for Cx3D is provided on the homepage of the Institute of Neuroinformatics of the University of Zurich and ETH Zurich (https://www.ini.uzh.ch/∼amw/seco/cx3d/Tutorial.pdf), where Cx3D has been developed [30]. CX3D is an open-source software written in Java for modelling all stages of corticogenesis in a 3D environment, based on the idea of the self-construction of the brain during development through autonomous agents. In CX3D, each cell is an independent entity which uses only locally available information. For the here presented floret generator, the autonomous agents are understood as the growth cones steering the developmental process. For our implementation in CX3D we use chiefly the NeuriteElement class to model the outgrowth of the neurites.

All final tests and implementations presented here were performed on a MacBook Pro with a 2.3 GHz Intel Core i7 processor, running Java 1.6.0 and MATLAB2016b. Version 6.9 of the MATLAB Parallel Computing Toolbox was employed for the MATLAB-based implementation of the model.

Parameter settings of the Genetic Algorithm

The here employed Genetic algorithm is an iterative procedures where in a first step an initial population - a set of possible solutions from the state space - is randomly generated. One set of initial parameter values are shown in S1 Table. For each such solution a fitness values is calculated according to the objective function given by the equation 5.1 in the main text.

Settings of the GA are presented in S2 Table. A detailed description of these settings can be found on https://www.mathworks.com/help/gads/genetic-algorithm-options.html

Results of the floret-generator model with confidence interval

Here, we present the results of the floret-generator based on different runs of the model. In particular, we run the floret-generator model 100 times with the optimized parameters presented in Table 3. Based on these realizations, we construct the 90% confidence interval of the generated segment-length distributions and of the individual statistics.

As before, we observe a generated unimodal segment-length distribution, which captures the unimodal shape of the biological segment-lengths (S1 Fig). The good fit of the floret-generator can also be observed for the individual statistics, which underline that the generated florets exhibit a similar morphological variability as the biological arborizations.

Results of the floret-generator with alternative parameters

Here, we present results of the floret-generator based on additional solutions of the optimization process. These parameters have been optimized by the Genetic Algorithm as previously described. To assess the robustness of the floret-generator, we use now, however, two different sets of parameters obtained by the optimization (S3 Table). As can be seen, the parameters differ only slightly from the best solution presented earlier (Table 3).

Based on these parmeters, we construct a 90% confidence interval from generated data from 100 realizations of the floret-generator. Again, we observe a good fit to the segment-length distribution of the biological data, as also previously observed (S3 Fig). A good fit can also be observed for the generated individual statistics of the florets. From these results, it can be concluded that the floret-generator model is robust to slight changes of the parameter values, and allows for a good fit to the biological data even when different solutions of the optimization process are being employed.

Results of the floret-generator without retraction

Here, we present the results of the floret-generator without retraction. As retraction is now excluded from the algorithmic growth process, the associated parameter ranges for optimization are set to zero. All other parameters, however, are optimized within the same range as the parameters of the model with retraction (see Table 3).

The optimized shape and scale parameters for growth are considerably higher in comparison to the model with retraction (S4 Table). The growth probability, and the parameters of the resource distribution, are, however, significantly smaller. Combined, these parameter values suggest that on average longer segments per florets are generated, but that these must be fewer in number to achieve an overall good fit to the biological data. This is apparent in the segment-length distribution where the characteristic lack of small segments observed in the biological data is not being matched (S4 Fig, Panel (A)).

Length distributions of intermediate and terminal segments of biological data

Previous studies of dendritic morphologies have revealed that terminal and intermediate segments differ with regard to the shape of their length distribution as well as with regard to their mean length [21, 23]. This observation makes it worthwhile to assess terminal and intermediate

segments separately also for the biological dataset at hand. In contrast to the cited dendritic studies, we find that while the segment-length distributions for terminal and intermediate segments are in both cases unimodal, right-skewed and long-tailed. Furthermore, we find that the mean segment-lengths are nearly identical, with a mean segment-length of 47.142 microns for the intermediate segments, and a mean segment-length of 47.237 microns for the terminal segments. A two sample ks-test can not reject the equality of the distributions ($D_{983,640} = 0.037$, $p > 0.4$). Hence, we conclude that the two kinds of segments can be described by the same set of growth rules.

Equivalence of the MATLAB and Cx3D segment-length distributions and individual statistics

We demonstrate empirically that the floret-generator can be ported into Cx3D by comparing the segment-length distributions and individual statistics generated by the algorithm described in Table 1 and implemented in MATLAB to those generated in the Cx3D environment as described in Section . For this comparison we generate 500 florets in each of the implementations, using the previously optimized parameters of the floret-generator presented in Table 3. In the MATLAB implementation the distribution of resource after bifurcations is decided by randomly sampling from the interval [b,1], with the bias parameter b being optimized by the GA. In contrast in Cx3D we model resource distribution as a function of extracellular gradients. As visible in S6 Fig, the segment-length distributions and the individual statistics of the two implementations show a nearly perfect fit, demonstrating a successful translation of the floret-generator into the Cx3D simulation environment. A two-sample Kolmogorov-Smirnov test fails to reject the null-hypothesis of equality for all the statistics of the MATLAB and the Cx3D ($D_{1544,1482} = 0.0229$, $p > 0.8$). The slight divergence between the two models can be attributed to the stochasticity of the modelled growth-processes.
