# Supplementary figures and images for: A generative growth model for thalamocortical axonal branching in primary visual cortex
Source: PLoS Comput Biol. 2020 Feb 13;16(2):e1007315. doi: 10.1371/journal.pcbi.1007315 (PMC7018004; doi:10.1371/journal.pcbi.1007315)

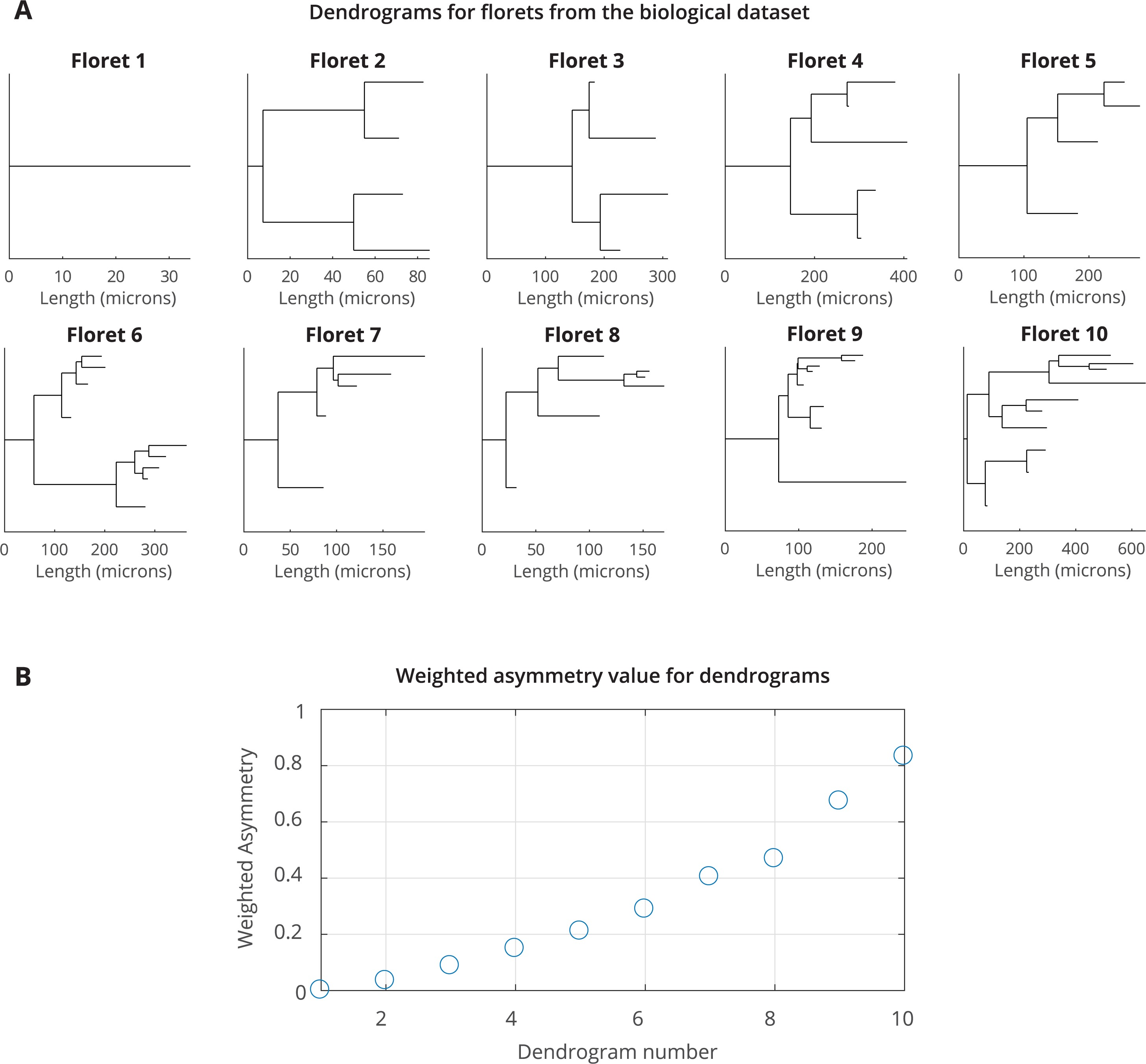

Supplement: S1 Fig — (A) Dendrograms of the florets from the biological dataset visualize the morphological diversity of the florets. These range from structures with only a single branch (top left) to morphologies with many branches of different lengths and topological orderings. (B) Weighted asymmetry values are plotted for the dendrograms presented in panel A, from left to right. Higher morphological complexity is reflected by an increased weighted asymmetry value, as this quantification considers both the length of the segments as well as their placement within the arborization pattern. (TIF) [file pcbi.1007315.s002.tif]

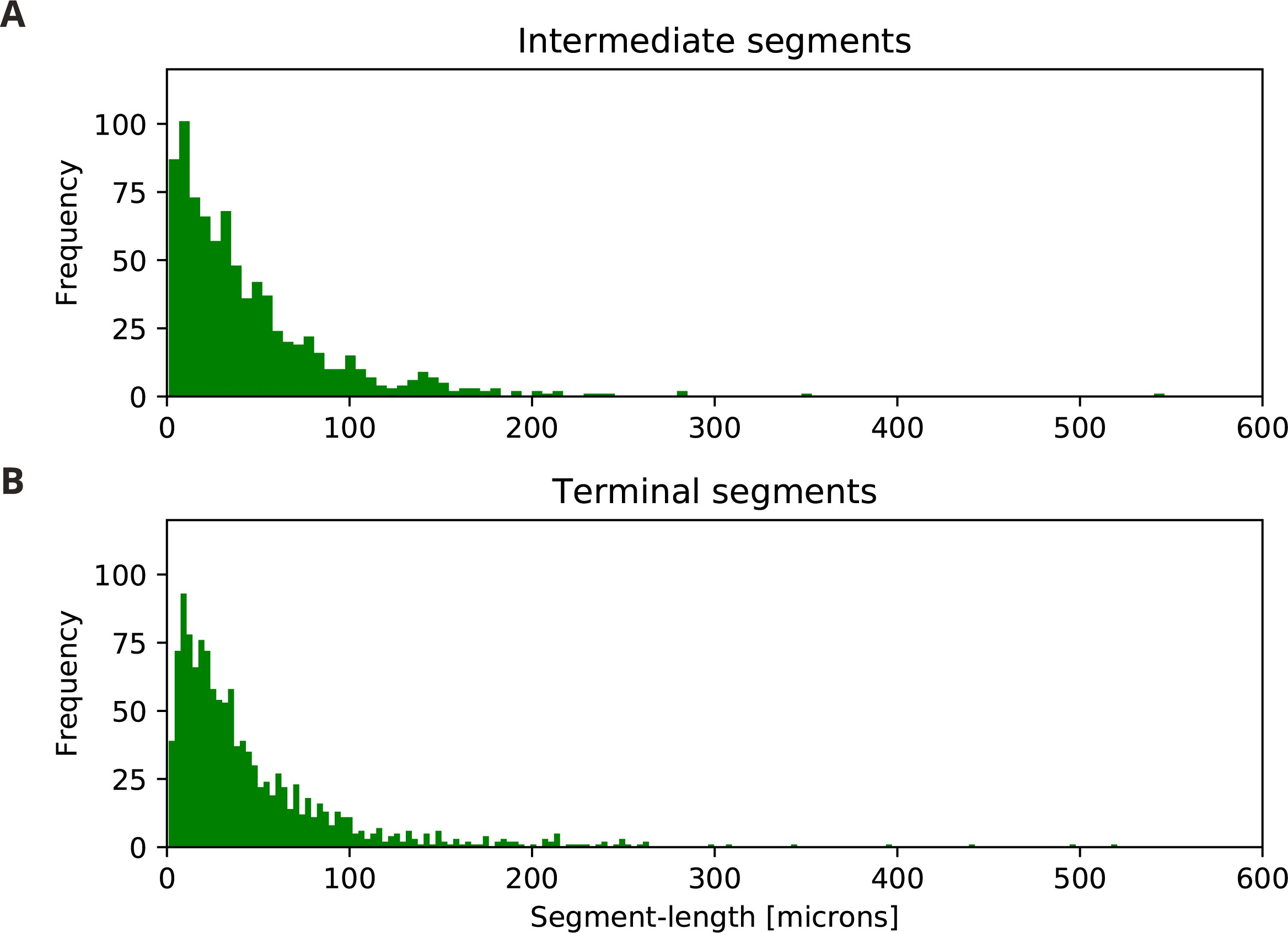

Supplement: S2 Fig — (A) The segment-length distributions of the terminal and (B) intermediate segments. Although the distributions are not entirely identical, they are in all cases unimodal, right-skewed and long-tailed. Additionally, the mean segment-lengths of intermediate and terminal segment are with 47.14 microns and 47.14 microns respectively remarkably similar. In addition, a two sample ks-test can not reject the equality of the distributions (p > 0.4). (TIF) [file pcbi.1007315.s003.tif]

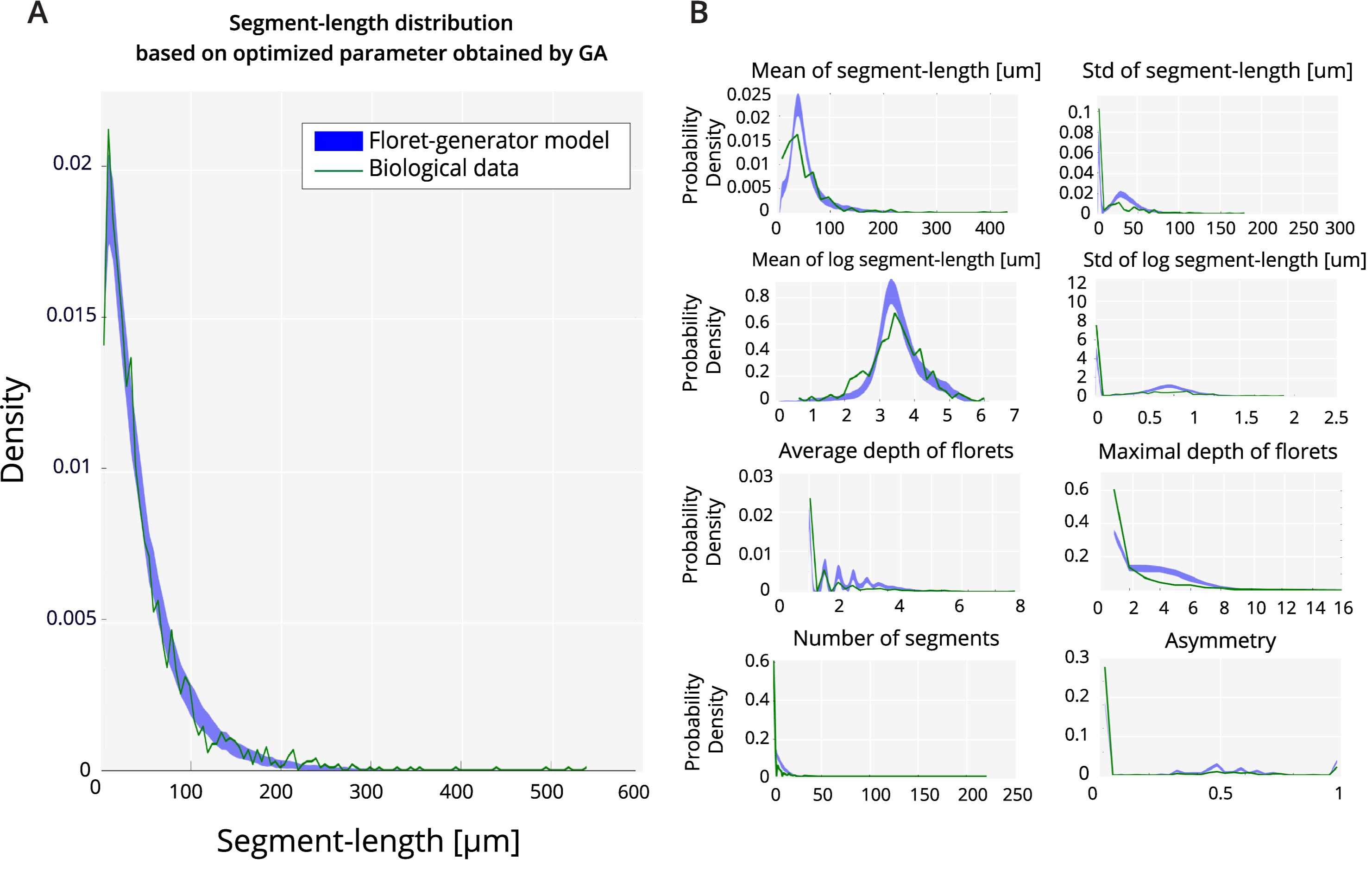

Supplement: S3 Fig — A 90% confidence interval is constructed from 100 realizations of the floret-generator with parameters optimized by the genetic algorithm (Table 3). (A) As visible, the segment-length distribution from the different runs replicates the unimodal distribution of the biological data closely. (B) We observe also a good fit of the generated data to the individual statistics of the biological florets, based on measures not employed for optimization. (TIF) [file pcbi.1007315.s004.tif]

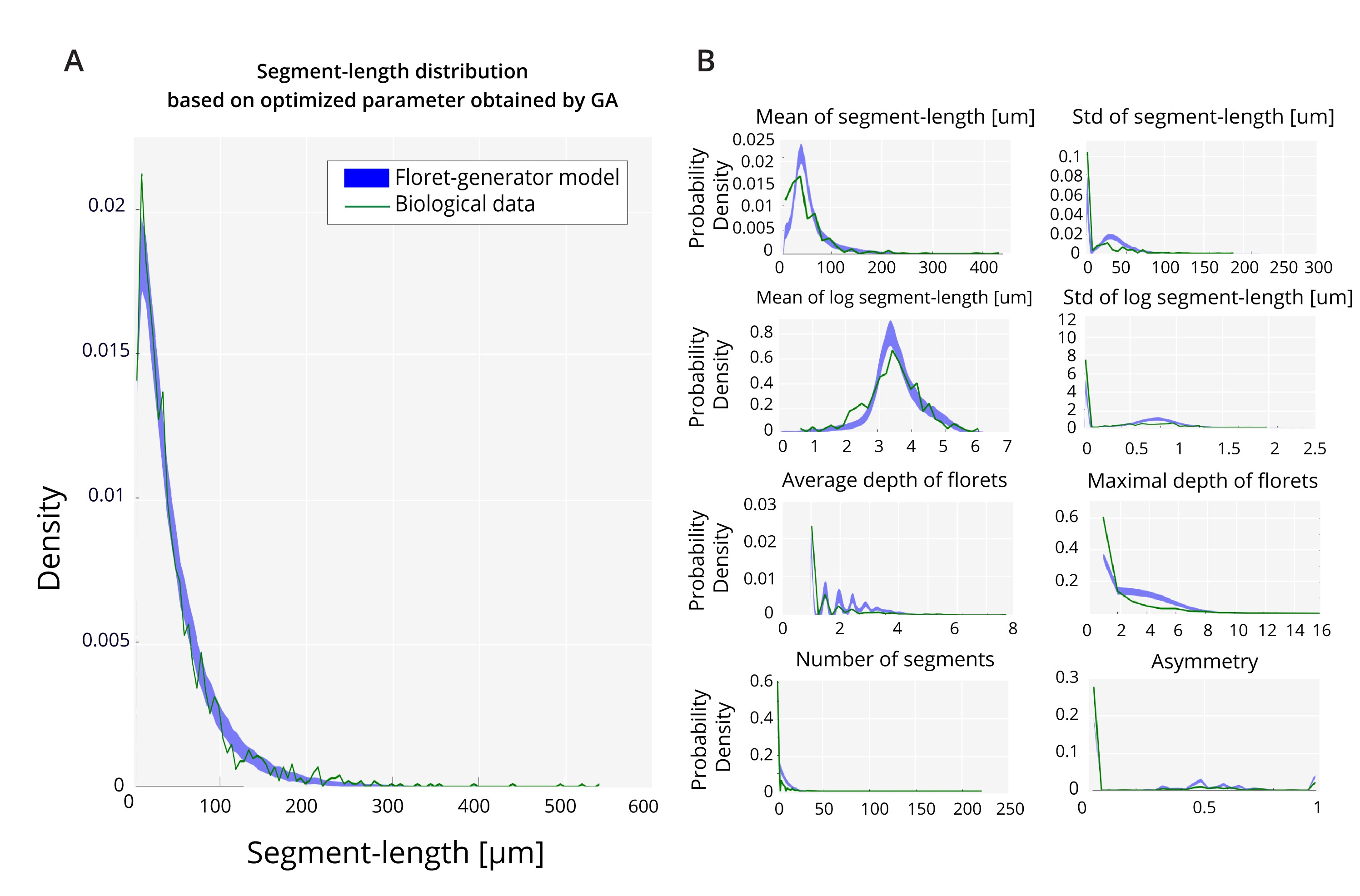

Supplement: S4 Fig — We construct a 90% confidence interval from the generated data based on a first alternative sets of optimized parameters and 100 realizations of the floret-generator. (A) Both the segment-length distribution, (B) as well as the individual statistics of the biological data, can be matched well based on the two parameter sets. We hence conclude that the good results obtained by the floret-generator are robust with respect to different solutions of the optimization process. (TIF) [file pcbi.1007315.s005.tif]

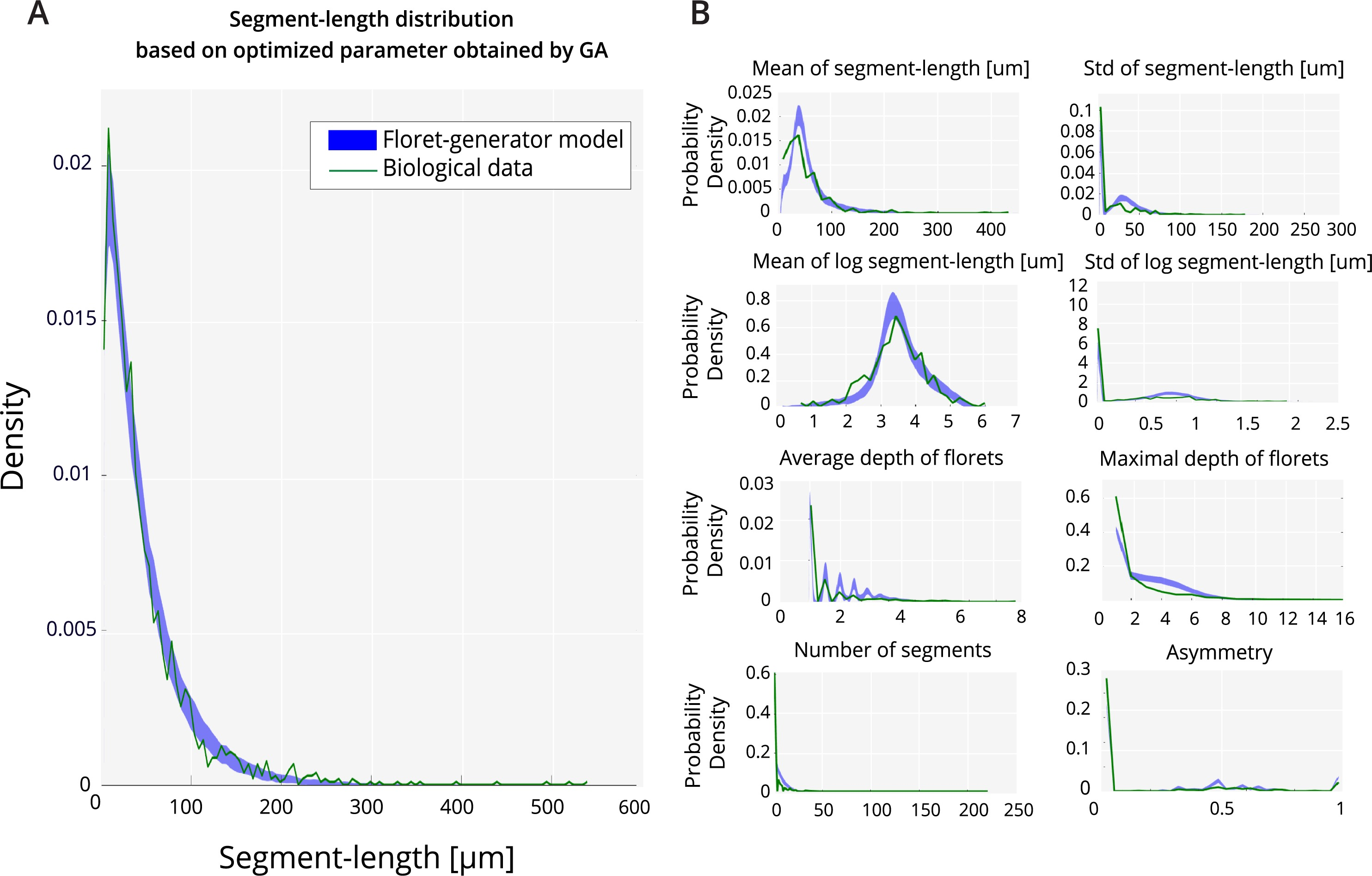

Supplement: S5 Fig — Here, we construct a 90% confidence interval from the generated data based on a second alternative sets of optimized parameters and 100 realizations of the floret-generator. (A) Both the segment-length distribution, (B) as well as the individual statistics of the biological data, can be matched well based on the two parameter sets. We hence conclude that the good results obtained by the floret-generator are robust with respect to different solutions of the optimization process. (TIF) [file pcbi.1007315.s006.tif]

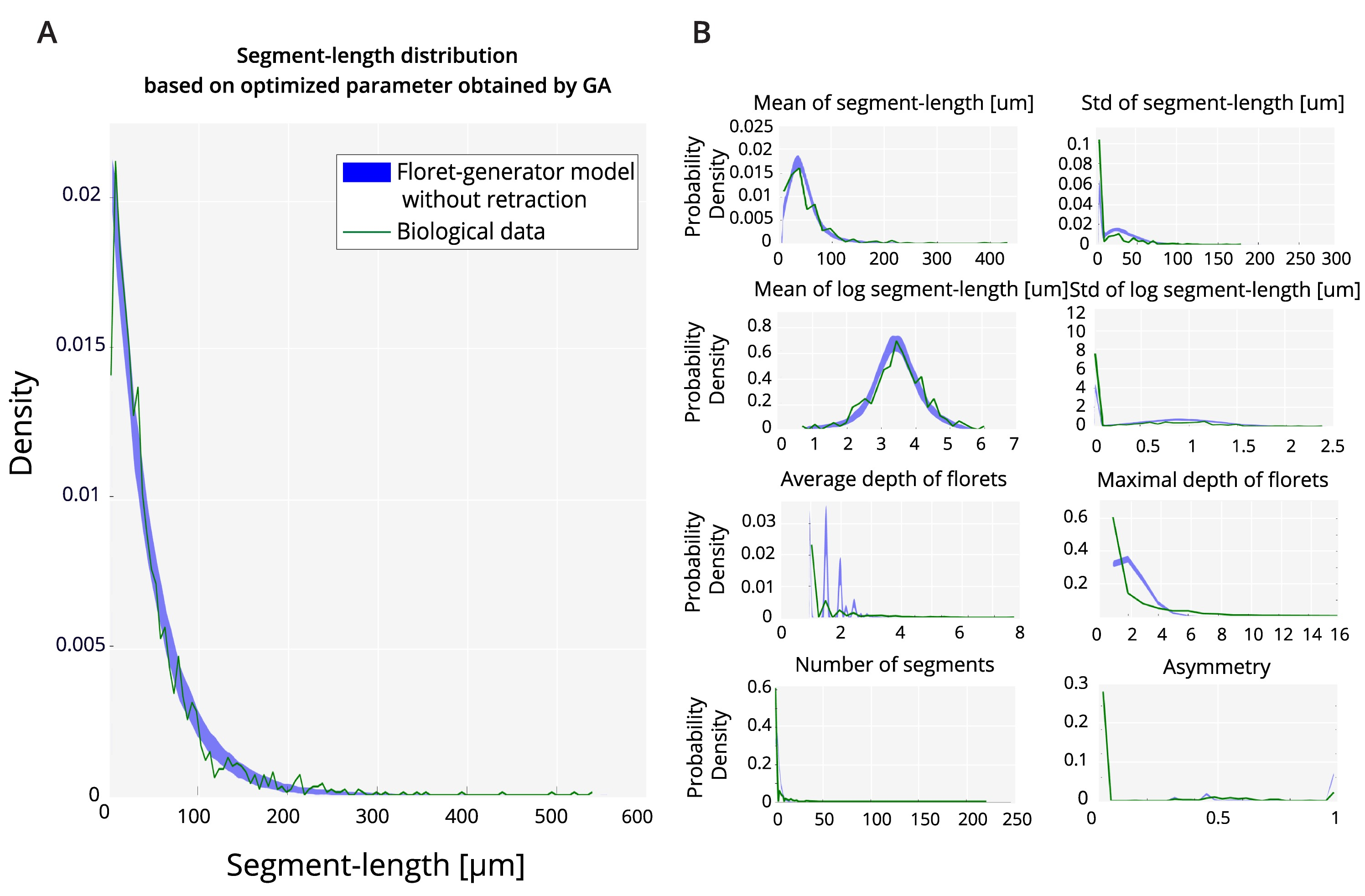

Supplement: S6 Fig — Panel (A) and (B) display the generated segment-length distribution of the floret-generator without retraction, based on the parameters optimized by the genetic algorithm. (A) The confidence interval based on 100 instantiations of the optimal parameters. The fit to the peak of the biological data is not optimal as the characteristic lack of small segments observed in the biological data can not be matched by this model. (B) The individual statistics of the floret-generator model without retraction, where the generated florets have typically only few segments. (TIF) [file pcbi.1007315.s007.tif]
