# Supplementary material for: A generative growth model for thalamocortical axonal branching in primary visual cortex
Source: PLoS Comput Biol. 2020 Feb 13;16(2):e1007315. doi: 10.1371/journal.pcbi.1007315 (PMC7018004; doi:10.1371/journal.pcbi.1007315)
Supplement: S1 Table — Parameters of the genetic algorithm employed for the optimization of the floret-generator. All other parameters are set to default values of the genetic algorithm from MATLAB’s Global Optimization Toolbox. PopulationSize: The number of solutions (also called ‘individuals’) in each generation. EliteCount: The fraction of the top-solutions passed on to the next generation. CreationFcn: The function creating the initial population for the genetic algorithm. SelectionFcn: The function which determines how the genetic algorithm chooses ‘parents’ for the next generation. ‘Parents’ are two solutions from which a new solution is generated. CrossoverFcn: The function specifies how the genetic algorithm combines two solutions to form a new solution for the next generation. MutationFcn: The function specifies how the genetic algorithm makes small random changes a solution in the population to create a new solution for the next generation. (PDF) [file pcbi.1007315.s009.pdf]

Table S1: Parameters of the Genetic Algorithm

Mathworks Function	Settings Floret Generator	Settings Galton-Watson
PopulationSize	60	60
EliteCount	0.1*(PopulationSize)	0.1*(PopulationSize)
CreationFcn	Uniform	Feasible population
SelectionFcn	Stochastic uniform	Stochastic uniform
CrossoverFcn	Scattered	Intermediate
MutationFcn	Gaussian	Adaptive Feasible

Parameters of the Genetic Algorithm employed for the optimization of the floret-generator. All other parameters are set to default values of the Genetic Algorithm from MATLAB's Global Optimization Toolbox. PopulationSize: The number of solutions (also called 'individuals') in each generation. EliteCount: The fraction of the top-solutions passed on to the next generation. CreationFcn: The function creating the initial population for the Genetic Algorithm. SelectionFcn: The function which determines how the Genetic Algorithm chooses 'parents' for the next generation. 'Parents' are two solutions from which a new solution is generated. CrossoverFcn: The function specifies how the Genetic Algorithm combines two solutions to form a new solution for the next generation. MutationFcn: The function specifies how the Genetic Algorithm makes small random changes a solution in the population to create a new solution for the next generation.
