# Supplementary material for: A generative growth model for thalamocortical axonal branching in primary visual cortex
Source: PLoS Comput Biol. 2020 Feb 13;16(2):e1007315. doi: 10.1371/journal.pcbi.1007315 (PMC7018004; doi:10.1371/journal.pcbi.1007315)
Supplement: S2 Table — The initial parameter values of the floret-generator of an initial population of the genetic algorithm. Parameter values are selected for the initial populations probabilistically within their individual ranges (for the ranges see main text Table 3). Over the following iterations, the genetic algorithm seeks an optimum of its search process by minimizing the divergence of segment-length and length-weighted asymmetry distributions between biological and generated data. (PDF) [file pcbi.1007315.s010.pdf]

Table S2: Initial parameter values of the Genetic Algorithm

Floret-generator parameters	Parameter description	Values of the initial population
Growth parameters	Elongation lengths [l_{growth}] are drawn from a gamma distribution with optimized shape and scale parameters in [0,100].	[g_{Sh}, g_{Sc}]
		[43.4, 1.6]
Retraction parameters	Retraction lengths [l_{retract}] are drawn from a gamma distribution with optimized shape and scale parameters in [0,100].	[r_{Sh}, r_{Sc}]
		[43.9, 2.2]
Resource parameters	For every floret, the amount of available resource r is drawn from a gamma distribution with optimized shape and scale parameters in [0,20].	[rs_{Sh}, rs_{Sc}]
		[10.8, 15.7]
Growth and retraction probabilities	The probability of growth and retraction are both sampled uniformly from [0,1].	[p_{growth}, p_{retract}]
		[0.8, 0.2]
The bias parameter	The bias parameter is optimized in the range of [0.5,1]. It determines the allocation of resource after bifurcation.	[b]
		[0.9]
The offset parameter	The offset parameter determines the initial length grown and is optimized within [1,2].	[os]
		[1.6]

The initial parameter values of the floret-generator of an initial population of the Genetic Algorithm. Parameter values are selected for the initial populations probabilistically within their individual ranges (for the ranges see main text Table S1). Over the following iterations, the genetic algorithm seeks an optimum of its search process by minimizing the divergence of segment-length and length-weighted asymmetry distributions between biological and generated data.
