# Supplementary material for: A generative growth model for thalamocortical axonal branching in primary visual cortex
Source: PLoS Comput Biol. 2020 Feb 13;16(2):e1007315. doi: 10.1371/journal.pcbi.1007315 (PMC7018004; doi:10.1371/journal.pcbi.1007315)
Supplement: S3 Table — Two alternative parameter sets of the floret-generator model optimized by the genetic algorithm. As visible, the parameters are remarkably similar to the best solution presented in Table 3. (PDF) [file pcbi.1007315.s011.pdf]

Table S3: Optimized parameters of the floret-generator

gSh	gSc	rSh	rSc	rsSh	rsSc	p_{growth}	$p_{\mathrm{retraction}}$	b	os
1.25	21.22	1.63	19.46	14.99	13.28	0.09	0.61	0.62	1.78
1.26	21.22	1.66	17.83	14.18	11.29	0.14	0.59	0.68	1.76

Two alternative parameter sets of the floret-generator model optimized by the Genetic Algorithm. As visible, the parameters are remarkably similar to the best solution presented in Table 3.
