# Supplementary material for: A generative growth model for thalamocortical axonal branching in primary visual cortex
Source: PLoS Comput Biol. 2020 Feb 13;16(2):e1007315. doi: 10.1371/journal.pcbi.1007315 (PMC7018004; doi:10.1371/journal.pcbi.1007315)
Supplement: S4 Table — The parameters of the floret-generator model without retraction as optimized by the genetic algorithm. Shape and scale parameters of the gamma distribution for growth are considerably higher as compared with the same parameters of the model with retraction. The growth probability and the parameters of the resource distribution are in contrast significantly smaller. (PDF) [file pcbi.1007315.s012.pdf]

Table S4: Optimized parameters of the floret-generator without retraction

gSh	gSc	rSh	rSc	rsSh	rsSc	p_{growth}	$p_{\text{retraction}}$	b	os
0.97	42.88	0.00	0.00	4.43	1.14	0.02	0.00	0.52	1.53

The parameters of the floret-generator model without retraction as optimized by the Genetic Algorithm. Shape and scale parameters of the gamma distribution for growth are considerably higher as compared with the same parameters of the model with retraction. The growth probability and the parameters of the resource distribution are in contrast significantly smaller.
